# Choice of transcripts and software has a large effect on variant annotation

**DOI:** 10.1186/gm543

**Published:** 2014-03-31

**Authors:** Davis J McCarthy, Peter Humburg, Alexander Kanapin, Manuel A Rivas, Kyle Gaulton, Jean-Baptiste Cazier, Peter Donnelly

**Affiliations:** 1 Department of Statistics, University of Oxford, South Parks Road, Oxford, UK; 2 Wellcome Trust Centre for Human Genetics, University of Oxford, Roosevelt Drive, Oxford, UK; 3 Department of Oncology, University of Oxford, Roosevelt Drive, Oxford, UK

## Abstract

**Background:**

Variant annotation is a crucial step in the analysis of genome sequencing data. Functional annotation results can have a strong influence on the ultimate conclusions of disease studies. Incorrect or incomplete annotations can cause researchers both to overlook potentially disease-relevant DNA variants and to dilute interesting variants in a pool of false positives. Researchers are aware of these issues in general, but the extent of the dependency of final results on the choice of transcripts and software used for annotation has not been quantified in detail.

**Methods:**

This paper quantifies the extent of differences in annotation of 80 million variants from a whole-genome sequencing study. We compare results using the RefSeq and Ensembl transcript sets as the basis for variant annotation with the software Annovar, and also compare the results from two annotation software packages, Annovar and VEP (Ensembl’s Variant Effect Predictor), when using Ensembl transcripts.

**Results:**

We found only 44% agreement in annotations for putative loss-of-function variants when using the RefSeq and Ensembl transcript sets as the basis for annotation with Annovar. The rate of matching annotations for loss-of-function and nonsynonymous variants combined was 79% and for all exonic variants it was 83%. When comparing results from Annovar and VEP using Ensembl transcripts, matching annotations were seen for only 65% of loss-of-function variants and 87% of all exonic variants, with splicing variants revealed as the category with the greatest discrepancy. Using these comparisons, we characterised the types of apparent errors made by Annovar and VEP and discuss their impact on the analysis of DNA variants in genome sequencing studies.

**Conclusions:**

Variant annotation is not yet a solved problem. Choice of transcript set can have a large effect on the ultimate variant annotations obtained in a whole-genome sequencing study. Choice of annotation software can also have a substantial effect. The annotation step in the analysis of a genome sequencing study must therefore be considered carefully, and a conscious choice made as to which transcript set and software are used for annotation.

## Background

The advent of accessible and relatively inexpensive high-throughput sequencing technology has resulted in extensive sequencing of whole human genomes or exomes in a research setting and seems likely to lead to an explosion of genomic sequencing in a clinical context. While there remain challenges in unambiguously determining an individual’s genome or exome sequence [1,2], our focus here is on the downstream interpretation of that sequence. Let us take as a starting point a specified list of positions, assumed to be correct, at which the nucleotides in the individual’s sequence differ from the human reference sequence. We will restrict our scope here to single nucleotide variants (SNVs) and short indels. A crucial step in linking sequence variants with changes in phenotype is variant annotation.

Variant annotation is the process of assigning functional information to DNA variants. There are many different types of information that could be associated with variants, from measures of sequence conservation [3] to predictions about the effect of a variant on protein structure and function [4-6]. Here we focus on the most fundamental level of variant annotation, which is categorising each variant based on its relationship to coding sequences in the genome and how it may change the coding sequence and affect the gene product.

The coding sequences of the genome are, broadly speaking, the genes: ‘gene’ has come to refer principally to a genomic region producing (through transcription) polyadenylated mRNAs that encode a protein [7]. We refer to these polyadenylated mRNAs as ‘transcripts’, although the term transcript can refer to any RNAs produced from the transcription of a genomic DNA sequence. Thus, there are non-coding transcripts that do not encode a protein, but nevertheless can have a function, for example in regulation. When considering transcripts in the context of genomic DNA sequences, a transcript is defined by its exons, introns and UTRs and their locations. Many separate transcripts may overlap any given position in the genome, and it is not uncommon for genes to have many different transcripts (or ‘isoforms’), of which they tend to express many simultaneously [8].

Our understanding of the protein-coding sequences in the genome is summarised in the set of transcripts we believe to exist. Thus, variant annotation depends on the set of transcripts used as the basis for annotation. The widely used annotation databases and browsers – ENSEMBL[9], REFSEQ[10] and UCSC[11] – contain sets of transcripts that can be used for variant annotation, as well as a wealth of information of many other kinds as well, such as ENCODE[12] data about the function of non-coding regions of the genome. A transcript set may therefore also contain information about regions of the genome that regulate expression of transcripts.

To annotate DNA variants we therefore require a set of transcripts that summarises our understanding of the genome. For each variant, we use a software tool to determine the likely effect of the variant based on the transcripts (or other genomic features) that overlap the variant’s position. One or more possible annotations for the variant can then be reported.

**Figure 1:**
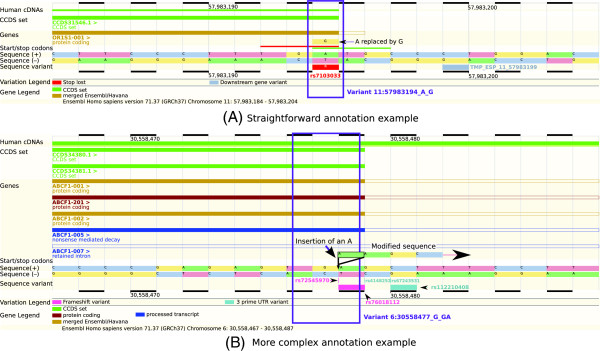
**Annotation examples.** These screenshots from the ENSEMBL web browser [40] show two examples of variant annotation. **(A)** The variant NC_000011.9:g.57983194A>G (rs7103033) is relatively straightforward to annotate. It is the final base of the final exon in both transcripts at this position (a CCDS transcript (green) and a ‘merged’ ENSEMBL/Havana (GENCODE) transcript (gold)). The final codon has changed from TGA (stop codon) to TGG (tryptophan), so this is unambiguously a stop-loss variant. Using the ENSEMBL transcript set, both ANNOVAR and VEP correctly annotate this variant as stop-loss. **(B)** The variant NC_000006.11:g.30558477_30558478insA (rs72545970) is more difficult to annotate. It is the penultimate base of the exon for all but one of the transcripts shown. It is a single-base insertion, so could be annotated as a frameshift variant. Then again, it is an insertion in a stop codon, so could be a stop-loss variant. In fact, the final codon, TGA (stop codon), remains TGA with this variant (insertion of a single base A), so it is actually a synonymous variant. ANNOVAR annotates it as frameshift insertion and VEP as stop-loss, when using ENSEMBL transcripts. Each browser image consists of several tracks, which provide base-resolution information about the DNA sequence. Two tracks, ‘Sequence (+)’ and ‘Sequence (-)’, show the DNA sequence on the forward and reverse strands, respectively. Above these, a track shows start and stop codons, and above that, several tracks indicate the presence and structure of different transcripts (labelled as ‘Genes’ and ‘CCDS set’; transcripts are read from left to right). The ‘hollowed-out’ parts of transcripts indicate non-coding sequences. Below the DNA sequence, the track ‘Sequence variant’ shows known sequence variants from dbSNP [17] and the 1000 Genomes Project [18]. The ‘Variation Legend’ and ‘Gene Legend’ provide more information about features shown in different colours in the browser. CCDS, Consensus Coding Sequence; UTR, untranslated region.

Variant annotation can be straightforward, as for the variant NC_000011.9:g.57983194A>G. Only two transcripts in the ENSEMBL transcript set, a Consensus Coding Sequence (CCDS) [13] transcript and a merged ENSEMBL/Havana (GENCODE) transcript [14,15], overlap the variant and the annotation of the transcript is the same regardless of which transcript is used (Figure 1A). This variant is unambiguously a stop-loss variant, as the final codon is changed from TGA (stop codon) to TGG (tryptophan) [9,16], and both of the software tools that we use for the present study correctly annotate this variant as stop-loss.

Frequently, however, variant annotation is more complex. Typical pipelines are not well suited for handling a variant that could have one consequence for one transcript and a different consequence for a different transcript. Even where a gene is relatively well defined and does not overlap other genes, we may have many transcripts (isoforms of the gene) to choose from, often supported by varying levels of evidence for their existence and structure. It is common for a gene to have multiple transcripts overlapping a given position in the genome, so given a set of transcripts a software tool has to choose which one to use. If it provides an annotation for the variant for each transcript, then the question becomes what annotation to report. If the software reports all possible annotations from all possible transcripts then the user must decide how to prioritise different, competing annotations, or how to integrate them into downstream analysis. This issue is further exacerbated in the uncommon case of a single variant affecting multiple genes (each of which likely has multiple transcripts). Current annotation tools vary in approaches to reporting consequences of a variant in multiple genes at once. Choice of the underlying set of transcripts used for annotation can give the user more control over transcript use. Transcript sets from different sources can have different characteristics. For example, both ENSEMBL and REFSEQ contain transcripts established from experimental evidence utilising automated annotation pipelines and manual curation, but their precise requirements for inclusion of transcripts differ. The result is that the ENSEMBL transcript set is larger than the REFSEQ set (see Additional file 1), but the REFSEQ transcript set is not simply a subset of the ENSEMBL transcript set.

Related to this issue is the fact that any given variant can have several plausible annotations, even when considering just a single transcript as the basis for annotation. Choosing the ‘best’ annotation is frequently not clear-cut, as in the case of the variant NC_000006.11:g.30558477_30558478insA, a single-base insertion at the end of an exon (Figure 1B). This variant could be annotated as a frameshift insertion in a coding sequence (which it is), or as a stop-loss variant (as it falls in a stop codon). In fact, the correct annotation is that this is a synonymous variant. In many cases we would be misled into thinking that the variant is a frameshift or stop-loss variant, and therefore be likely to assume it has a functional effect and include it in any list of variants of interest for further investigation. Indeed, one of the software tools used for this study reports a frameshift insertion annotation and the other a stop-loss annotation for this variant, when using ENSEMBL transcripts. In this example there seems to be a single best annotation, but many cases are more ambiguous, with several equally valid possible annotations. The software tool must make some sort of choice in such cases as to which annotation to report for the variant (and transcript used). There are many other annotation tools available (for example, Mutalyzer 2 [19], VAT [20], VAAST 2.0 [21], GATK VariantAnnotator [22] and SnpEff [23]), which will have better or worse performance for certain variants, but here we want to make the more general point using two very widely used annotation tools, that there is a large degree of discrepancy between any two annotation tools, and researchers need to be aware of this when choosing a tool and conducting analysis.

A third major issue complicating variant annotation is the question of how to deal with genes and pseudogenes. We have widely varying levels of information available for different genes. Should we treat variants in well-characterised genes in the same way as those in pseudogenes or non-genic regions of the genome? There is not currently a clear solution to this issue, although distinctions are usually made between annotations given from coding and non-coding transcripts. Again, careful choice of transcript set used for annotation can help.

Although there are many complications for variant annotation, we identify two major components: 

1. Transcript set: a summary of information about genomic features, particularly the structure of transcripts (sequence and locations of exons, introns, UTRs and regulatory regions), used as the basis for determining the likely functional consequence of a variant.

2. Software tool: a piece of software that when given a particular variant can query a transcript set and return the functional annotation (or possibly annotations) of that variant. An annotation tool uses a particular algorithm applied to a given set of transcripts for annotating variants.

We examine the effects of fixing one and then the other on a set of over 80 million SNVs and short indels from a large clinical sequencing project (see Methods). ANNOVAR[24] is a popular annotation software tool, so we compare the results from ANNOVAR when used with the REFSEQ and ENSEMBL transcript sets. We also compare the annotation results from ANNOVAR and another popular annotation tool, VEP[25], the Variant Effect Predictor tool from ENSEMBL, when using the ENSEMBL transcript set and characterise the sorts of differences in annotation between the two tools and the apparent errors that ANNOVAR and VEP tend to make in annotation. Beyond issues specific to these particular transcript sets and software tools, we consider good practice for whole-genome annotation and problems that are yet to be solved.

## Methods

### Data generation

The data used in this paper come from the WGS500 Project, a collaboration between the University of Oxford, Oxford Biomedical Research Centre and Illumina, Inc, to sequence 500 genomes of clinical relevance. Samples were accepted from patients where positive findings would have immediate clinical translational relevance in terms of clinical diagnosis, prognosis, genetics counselling and reproductive options, or treatment selection. As seen in some of the published studies that participated in the WGS500 project [26-32], this large umbrella project consists of many smaller sub-projects, focusing on particular diseases. All patients in this study gave written informed consent. The relevant research ethics committee (REC) reference numbers are: Central Oxfordshire Research Ethics Committee (05/Q1605/88), Hammersmith and Queen Charlotte’s and Chelsea REC (06/Q0406/151), NRES Committee South Central—Oxford B (12/SC/0381), NRES Committee South Central—Southampton A (12/SC/0044), NRES Committee North West—Haydock (03/0/97 version 3), NRES Committee Yorkshire & The Humber—South Yorkshire (10/H1310/73), Oxfordshire Research Ethics Committee (06/Q1605/3), Oxfordshire Research Ethics Committee B (04.OXB.017; 09/H0605/3) and Oxfordshire Research Ethics Committee C (09/H0606/74; 09/H0606/5), Riverside Research Ethics Committee (09/H0706/20) and Southampton and South-West Hampshire REC A (06/Q1702/99). The research conformed to the Helsinki Declaration and to local legislation.

We focus here on whole genomes of 276 individuals sequenced as part of the WGS500 project. The samples included 80 patients with immune disease, 151 individuals from Mendelian disease studies (primarily parent–child trios) and 45 germ-line DNA samples from cancer patients. The sequencing was conducted using 100-bp paired-end protocols on either the Illumina HiSeq 2000 instrument [33] or the Illumina HiSeq 2500 in standard mode [34], with a mixture of v2.5 and v3.0 chemistries, to at least 25 × average coverage. Sequence reads were generated using the Illumina Off-Line Basecaller (v1.9.3) [35] and mapped to the human reference genome GRCh37d5/hg19d5 using Stampy, predominantly versions 1.0.12_(r975) and 1.0.13_(r1160) [36]. Picard (picard-tools v1.67) was used to merge data and de-duplicate merged BAM files [37]. Variants were called from the aligned sequence reads using Platypus, version 0.1.9 [38]. The raw data for annotation are VCF (variant call format) files [39] containing information about the called variants.

In total, 80,995,744 unique variant calls were obtained from 276 individual genomes in the fifth freeze of the project’s data, and merged into a preliminary union file. We compare functional annotations for 80,981,575 variants from the preliminary union file using transcript sets from different genome annotation databases and different annotation software tools, restricting ourselves to the set of variants for which an annotation was obtained using at least one transcript set or software tool.

### Variant annotations

Variant annotations were obtained using the software tool ANNOVAR(version 2013Feb21), using both the REFSEQ (release 57, January 2013) and ENSEMBL (version 69, October 2012) transcript sets [10,40]. We used the default transcript sets from REFSEQ and ENSEMBL. REFSEQ records are selected and curated from public sequence archives, so a REFSEQ record represents a synthesis, by a person or group, reducing the redundancy in the database. The REFSEQ database does not contain all possible (or even all observed) transcripts or gene models, but those that it does annotate feature strong evidence for their existence, structure and (possibly) function. Of a total of 105,258 human transcripts in REFSEQ release 57, 41,501 were used by ANNOVAR in the reported annotations for the variants in this study.

Similarly, ENSEMBL provides genome resources for chordate genomes with a particular focus on human genome data. ENSEMBL makes available substantial and diverse transcript information, including the CCDS [13,41], Human and Vertebrate Analysis and Annotation (HAVANA) [42], Vertebrate Genome Annotation (Vega) [43], ENCODE data [12] and the GENCODE gene and transcript sets [15]. There are 208,677 transcripts in ENSEMBL version 69, of which 115,901 were used in reported annotations for this comparison.

A broad interpretation of splicing regions was used for ANNOVAR annotations, so that all variants within six bases of an intron/exon boundary would fall into ANNOVAR’s splicing annotation category. ANNOVAR returns a single annotation for each variant. If there are several relevant transcripts for a particular variant, then ANNOVAR will return the annotation with the most severe consequence according to its rules of precedence.

Variant annotations were also obtained using version 2.7 of ENSEMBL’s VEP, based on the ENSEMBL version 69 transcript set. As VEP returns all possible annotations for each variant (given the transcripts present at each variant’s location in the genome), we prioritised annotations using a common-sense ranking of the ‘severity’ of the consequence of the variant (Additional file 1: Table S3) to make the VEP annotation results directly comparable with those from ANNOVAR. This prioritisation for consequences from VEP is just one possible way to prioritise variants and this subjectivity could affect the extent of matching between annotations from ANNOVAR and VEP. The most severe consequence for each variant was reported and compared to the ANNOVAR results.

### Comparisons of variant annotations

We compared results across all annotation categories for the REFSEQ/ENSEMBL comparison. A comparison table (union_rfs_ens_comparison.tab[44]), was produced with a custom Perl[45] script from VCF files containing ANNOVAR annotations when using REFSEQ and ENSEMBL transcripts and gene information for the transcript(s) used for each annotation. ANNOVAR reports only the ‘most damaging’ annotation, but can return transcript information for all transcripts that would give the annotation reported. Subsequent statistical analysis was done in R version 2.15.0 [46].

For the comparison of ANNOVAR and VEP we focused on exonic variants (and especially loss-of-function (LoF) and nonsynonymous variants) for the ANNOVAR/VEP comparison as these are currently of the greatest interest in the majority of annotation applications in whole-genome sequencing (WGS) studies. A VCF file containing all variants for comparison with annotations from ANNOVAR was processed to obtain VEP annotations, and the results were processed with a custom Python[47] script to create a table of variants (ANV_VEP_ens_comparison_best_annos.tab[44]). The table provides annotation results obtained using ENSEMBL transcripts with ANNOVAR and VEP, and information on transcripts used. The table was then analysed with R. We used the ENSEMBL Web Browser (archive version of ENSEMBL 69) [40] and the UCSC Genome Browser [48] to inspect sets of variants identified to be of particular interest by comparing annotations using the DNA sequence and other information available in the browser. Source code for the analyses described here is available in the repository containing the data, along with a ‘README’ file that provides more details about the data and source code files.

### Categories of variant annotations

To present, explain and discuss the results of our comparisons we need to introduce the different types of annotations produced by the different annotation tools. We define four high-level categories of variants that are of particular interest for many functional studies: 

1. **Putative LoF variants:** variants that are likely to cause a gene product to be subject to nonsense-mediated decay and result in lost (or impaired) function of the gene. We include in this high-level category frameshift deletions, frameshift insertions, and stop-gain, stop-loss and (most) splicing variants. Where finer resolution splicing categories are available (for example from VEP and some other annotation tools), we classify variants in splice acceptor and splice donor sites as LoF and other splicing variants as generically exonic (defined below). Annovar does not provide subcategories of splicing variants, so for our study we include all splicing variants in the LoF high-level category.

2. **Nonsynonymous and missense variants:** variants in exons that change the amino acid sequence encoded by the gene (but are not LoF), including single-base changes and nonframeshift indels. For this study we include VEP’s ‘splice_region_variant’ annotation in the missense high-level category as this reflects the fact that general splice region variants are usually of a similar level of interest as canonical missense variants.

3. **Synonymous variants:** variants located in exons that do not change the translated amino acid sequence.

4. **Exonic variants:** variants that fall anywhere in exons or splicing regions, so this includes all variants in the LoF, nonsynonymous and synonymous high-level categories above.

The exact terms used to denote annotation categories differ between ANNOVAR and VEP, but the correspondence in terms is almost always clear (Additional file 1: Table S4). There are three exonic categories used by VEP (initiator codon variant, stop retained variant and other coding) for which there is no direct equivalent among the ANNOVAR categories.

## Results and discussion

### Same annotation tool, different transcript sets

The comparison of annotation results from ANNOVAR using either the REFSEQ or ENSEMBL transcript sets shows that the choice of transcript set has a large effect on the ultimate variant annotations. Across all 80 million variants there is an overall match rate of 85%. However, the matching annotation rate is 44% for LoF variants, the set of variants of most interest for biological and medical studies. The match rate is also substantially lower than the overall match rate for variants in non-coding RNA and UTR regions, but there is better agreement for exonic and intronic variants. This observation accords with what we would expect: in areas of the genome where more is known about the protein-coding structure of the sequence, the annotations when using the two transcript sets agree more closely.

There are 590,893 variants given exonic annotations by ANNOVAR using REFSEQ or ENSEMBL (or both), of which 488,113 (83%) had precisely matching annotations when using the two different transcript sets (Table 1). The breakdown of matching variants by annotation reveals annotation categories showing greater and lesser difference when using REFSEQ or ENSEMBL. The extent of annotation matching is also summarised by high-level category: LoF, LoF and missense (nonsynonymous), exonic and all annotated.

**Table 1:** **Same software, different transcripts:**REFSEQ **vs **ENSEMBL **by **ANNOVAR **annotation category**

	**REF+ENS**	**REF**	**ENS**	**Match**	**REF match**	**ENS match**	**Overall match**
					**rate (%)**	**rate (%)**	**rate (%)**
stopgain_SNV	15,835	14,183	14,960	13,308	93.83	88.96	84.04
frameshift_insertion	6,980	5,298	6,495	4,813	90.85	74.10	68.95
frameshift_deletion	7,491	4,547	7,380	4,436	97.56	60.11	59.22
stoploss_SNV	946	503	906	463	92.05	51.10	48.94
splicing	47,878	14,154	45,839	12,115	85.59	26.43	25.30
frameshift_substitution	1,960	195	1,947	182	93.33	9.35	9.29
nonsynonymous_SNV	321,669	291,898	315,592	285,821	97.92	90.57	88.86
nonframeshift_insertion	3,506	2,888	2,844	2,226	77.08	78.27	63.49
nonframeshift_deletion	5,136	3,321	4,963	3,148	94.79	63.43	61.29
nonframeshift_substitution	933	226	843	136	60.18	16.13	14.58
synonymous_SNV	178,559	167,561	172,463	161,465	96.36	93.62	90.43
UTR3	724,802	574,255	622,441	471,894	82.17	75.81	65.11
UTR5	177,832	94,545	162,684	79,397	83.98	48.80	44.65
UTR5_UTR3	2,183	292	2,092	201	68.84	9.61	9.21
ncRNA_intronic	8,992,009	2,113,428	8,244,441	1,365,860	64.63	16.57	15.19
ncRNA_exonic	654,098	140,303	597,947	84,152	59.98	14.07	12.87
ncRNA_UTR3	53,379	10,712	47,133	4,466	41.69	9.48	8.37
ncRNA_UTR5	10,683	1,989	9,444	750	37.71	7.94	7.02
ncRNA_splicing	13,931	1,051	13,562	682	64.89	5.03	4.90
ncRNA_UTR5_ncRNA_UTR3	107	1	106	0	0.00	0.00	0.00
intronic	29,289,037	26,805,864	27,743,749	25,260,576	94.24	91.05	86.25
intergenic	50,305,202	49,797,113	41,307,708	40,799,619	81.93	98.77	81.10
downstream	991,811	474,684	840,376	323,249	68.10	38.46	32.59
upstream	910,818	440,728	762,664	292,574	66.38	38.36	32.12
upstream_downstream	53,608	15,621	47,293	9,306	59.57	19.68	17.36
unknown	11,205	6,215	5,703	713	11.47	12.50	6.36
ALL LOF	81,090	38,880	77,527	35,317	90.84	45.55	43.55
ALL LOF and MISSENSE	412,334	337,213	401,769	326,648	96.87	81.30	79.22
ALL EXONIC	590,893	504,774	574,232	488,113	96.70	85.00	82.61
ALL	80,981,575	80,981,575	80,981,575	69,181,552	85.43	85.43	85.43

This table summarises the number of annotations that match between the REFSEQ and ENSEMBL results for each category of annotation. It shows the number of variants given each type of annotation when using (i) either REFSEQ or ENSEMBL (‘REF+ENS’; union), (ii) REFSEQ (‘REF’) and (iii) ENSEMBL (‘ENS’). It also shows the number of variants that have matching annotations (i.e. the same annotation when using both transcript sets; intersection) and the match rate for each transcript set, which expresses the proportion of matching annotations for an annotation term relative to the total number of annotations in the category from the particular transcript set, as a percentage. The final column shows the ‘Overall match rate’, which is the percentage of the variants with a given annotation when using either REFSEQ or ENSEMBL (‘REF+ENS’) that have a matching annotation when using the two transcript sets. Categories are loosely ordered by the severity of effect, with LoF annotations listed before nonsynonymous, synonymous, non-exonic categories and so on. Within each loose group, categories are sorted in descending order of overall matching rate. The bottom four rows show the total degree of matching across all putative loss-of-function (LoF) categories, all LoF and missense categories, all exonic categories and, finally, all categories.

**Figure 2:**
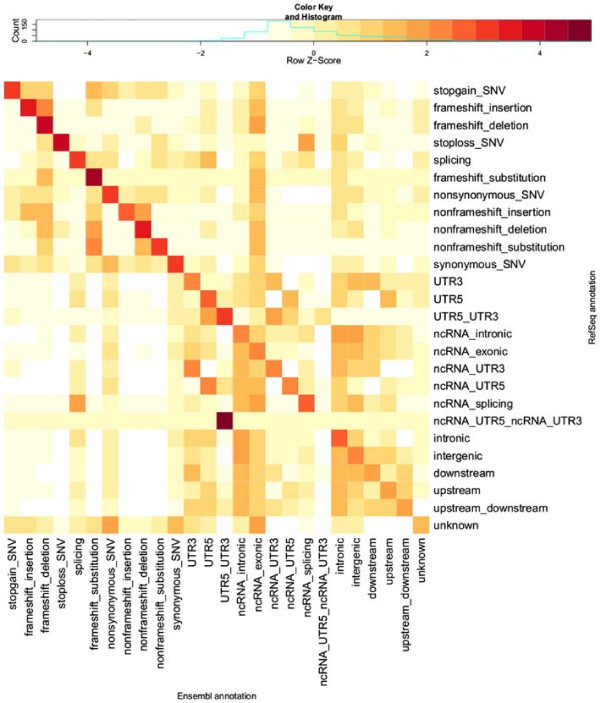
REFSEQ**-normalised heatmap of annotation comparison.** This heatmap shows scaled numbers of variants (log10 transformation with offset of 1 applied) for all different combinations of ANNOVAR categories of annotations when using the ENSEMBL transcript set (columns) and REFSEQ transcript set (rows). Values are Z-scaled (mean-centred, divided by standard deviation) by row (each row is scaled separately; contrast with Figure 3). The key above the heatmap shows the values indicated by different colours. This row-normalised heatmap allows us to see which categories of annotation are over-represented (relative to the total number of variants in the column/category) in the ENSEMBL annotations for each category (i.e. row) of REFSEQ annotation. Ideally, all of the dark red squares would lie on the diagonal, with white squares on the off-diagonals, indicating complete agreement in the annotations from the two transcript sets. Compare with Additional file 1: Table S1, which provides the numbers used for this heatmap. Categories are ordered as per Table 1.

**Figure 3:**
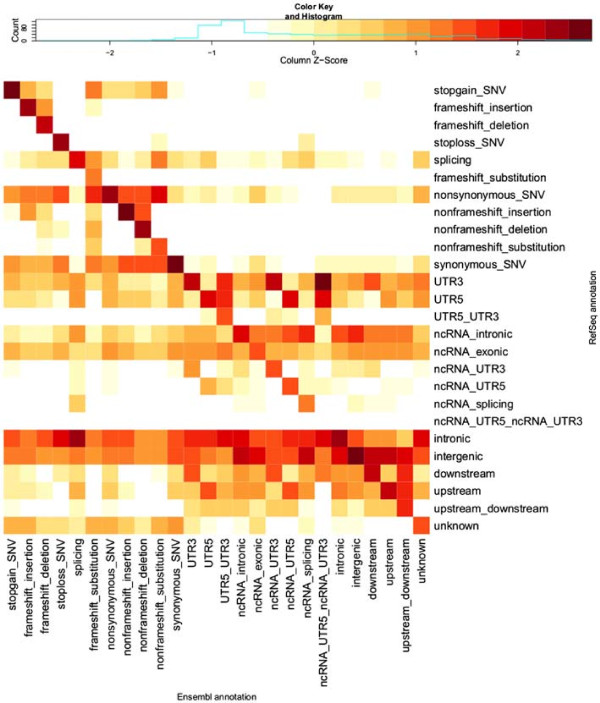
ENSEMBL**-normalised heatmap of annotation comparisons.** This heatmap shows scaled numbers of variants (log10 transformation with offset of 1 applied) for all different combinations of ANNOVAR categories of annotations when using the ENSEMBL transcript set (columns) and REFSEQ transcript set (rows). Values are Z-scaled (mean-centred, divided by standard deviation) by column (each column is scaled separately; contrast with Figure 2). The key above the heatmap shows the values indicated by different colours. The column-normalised heatmap allows us to see which categories of annotation are over-represented (relative to the total number of variants in the column/category) in the REFSEQ annotations for each category (i.e. column) of ENSEMBL annotation. Ideally, all of the dark red squares would lie on the diagonal, with white squares on the off-diagonals, indicating complete agreement in the annotations when using the two transcript sets. Compare with Additional file 1: Table S1, which provides the numbers used for this heatmap. Categories are ordered as per Table 1.

Visual comparison of transcript sets using REFSEQ- and ENSEMBL-normalised counts of variants with each combination of annotation terms from the two transcript sets highlights patterns in the differences in annotations provided by REFSEQ and ENSEMBL (Figures 2 and 3). By ‘REFSEQ-normalised’, we mean that for each annotation term we consider all of the variants given that annotation using REFSEQ across all annotations using ENSEMBL and then normalise the count for each ENSEMBL annotation within the REFSEQ annotation by subtracting the mean number of counts per ENSEMBL annotation and dividing by the standard deviation. We do this independently for each REFSEQ annotation term. To obtain ‘ENSEMBL-normalised’ values we do precisely the same thing, but exchange the roles of the ENSEMBL and REFSEQ annotations. Thus, for a given annotation term for a given transcript set, we can see the relative breakdown of annotations obtained when using the other transcript set. The REFSEQ-normalised values (Figure 2) show good agreement for indels (frameshift and nonframeshift), stop-gain, stop-loss and nonsynonymous variants, that is, a large proportion of variants given a particular annotation when using REFSEQ also get that annotation when using ENSEMBL. The agreement is not as good for synonymous and splicing variants, but we observe that variants given an exonic annotation when using REFSEQ usually get the same annotation when using ENSEMBL. Looking at ENSEMBL-normalised values (Figure 3), we see generally lower matching rates. Agreement is good for variants called stop-gain, nonframeshift, nonsynonymous and synonymous by ENSEMBL, but variants annotated as frameshift, stop-loss and splicing are frequently given a different annotation when using REFSEQ.

The asymmetry in the differences in annotations between REFSEQ and ENSEMBL is striking. We see many more exonic annotations, across all LoF, nonsynonymous and synonymous categories, when using ENSEMBL transcripts (Table 1 and Additional file 1: Table S1). There are several thousand variants that are called exonic by ENSEMBL and yet are called as intergenic, intronic or in a non-coding RNA by REFSEQ. Conversely, there are only a few hundred exonic variants from REFSEQ that are annotated as intergenic, intronic or in non-coding RNA according to ENSEMBL. Using ENSEMBL here would gain over 2,000 frameshift indels and over 1,000 stop-gain/stop-loss variants compared with using REFSEQ, which all LoF variants of substantial interest for follow-up. This asymmetry is not surprising when we consider the composition of the two transcript sets. The REFSEQ set contains 105,258 human transcripts in release 57, for which the protein-coding sequences cover approximately 1.07% of the genome (34 Mb). ANNOVAR actively used 41,501 of these transcripts for annotation of this set of variants. The ENSEMBL version 69 set contains 208,677 transcripts (192,635 on chromosomes 1 to 22, X and Y, excluding patches and alternate loci), covering approximately 28% of the genome (892 Mb), including introns. The protein-coding sequences in the ENSEMBL transcript set cover approximately 1.12% of the genome (35 Mb). Of these transcripts, 115,091 were actively used for the annotation of this set of variants, including the set of 92,776 transcripts containing protein-coding sequences.

This extent of discrepancy in annotations can be partially explained by the fact that a high proportion of REFSEQ transcripts have an equivalent or highly similar transcript in ENSEMBL, but in the other direction there are many transcripts in ENSEMBL that do not appear to have a similar transcript in REFSEQ. ANNOVAR reports the most severe consequence for a variant across all transcripts present at that position in the genome, so with more transcripts available when using ENSEMBL there is an elevated chance of finding a more severe consequence for one of the ENSEMBL transcripts. Examples of variants with striking differences in annotation help to characterise the sorts of differences seen (Additional file 1: Figures S1 to S8). We saw no significant differences in annotation agreement rates across different variant frequencies (Additional file 1: Table S5a).

### Same transcript set, different annotation tools

We also investigate the extent to which using different software tools influences the final annotations. Here we compare annotations from ANNOVAR and VEP using the ENSEMBL transcript set, focusing on exonic annotation categories. We look at the rate of ‘exactly matching’ annotations and the rate of ‘category matching’ annotations. We refer to an exact match when the annotations from both software tools are exactly equivalent given the annotation terms used by the two tools, for example both tools annotate a variant as frameshift. By category match, we mean that annotations from both software tools are in the same high-level category of LoF, missense or synonymous and other coding (with high-level categories defined in Additional file 1: Table S4). So if a variant received an annotation of frameshift from one tool and stop-gain from the other we would designate this as a category match as both are LoF annotations. Overall, we see only a small difference in matching rates when we consider category matches as opposed to exact matches, with category matching rates approximately 1% higher than exact matching rates (Table 2).

**Table 2:** **Same transcripts, different software:**ANNOVAR **and **VEP **annotations for exonic variants**

	**ANV + VEP**	**ANV**	**VEP**	**Exact**	**Category**	**ANV match**	**VEP match**	**Overall**	**Overall**
				**match**	**match**	**rate (%)**	**rate (%)**	**category match**	**exact match**
								**rate (%)**	**rate (%)**
LOF total	104,915	77,527	96,761	68,284	69,373	88.08	70.57	66.12	65.09
Frameshift	19,021	15,822	16,685	13,486	-	85.24	80.83	-	70.90
Stop gained	16,758	14,960	16,146	14,348	-	95.91	88.86	-	85.62
Stop lost	1,113	906	1,077	870	-	96.03	80.78	-	78.17
All splicing	69,112	45,839	62,853	39,580	-	86.35	62.97	-	57.27
MISSENSE total	350,806	324,242	347,752	318,056	321,188	98.09	91.46	91.56	90.66
Inframe indel	9,455	8,650	6,600	5,795	-	66.99	87.80	-	61.29
Missense	343,284	315,592	339,953	312,261	-	98.94	91.85	-	90.96
Initiator codon	1,199	0	1,199	0	-	-	0.00	-	0.00
SYNONYMOUS and									
OTHER CODING total	182,120	172,463	175,483	165,643	165,826	96.05	94.39	91.05	90.95
Synonymous	181,873	172,463	175,053	165,643	-	96.05	94.62	-	91.08
Stop retained	203	0	203	0	-	-	0.00	-	0.00
Other coding	227	0	227	0	-	-	0.00	-	0.00
ALL LOF	104,915	77,527	96,761	68,284	69,373	88.08	70.57	66.12	65.09
ALL LOF and MISSENSE	455,721	401,769	444,513	386,340	390,561	96.16	86.91	85.70	84.78
ALL EXONIC	637,841	574,232	619,996	551,983	556,387	96.13	89.03	87.23	86.54

This table summarises the number of annotations that match between the ANNOVAR and VEP results (when using ENSEMBL transcripts) for each exonic category of annotation. It shows the number of variants given each type of annotation by when using (i) either ANNOVAR or VEP (‘ANV+VEP’; union), (ii) ANNOVAR (‘ANV’) and (iii) VEP (‘VEP’). It also shows the number of variants that have exact matching annotations (i.e. exactly the same annotation from both tools; intersection), and category-matching annotations (i.e. annotations from the two tools in the same high-level category – LoF, missense, synonymous and other coding – even if not an exact match). Columns six and seven show the match rate for each tool, which gives the percentage of matching annotations for an annotation term from ANNOVAR and VEP, respectively, relative to the total number of annotations in the category from the particular software tool. Column eight gives the percentage of variants with annotations from ANNOVAR and VEP in the same high-level category (overall category match rate). Column nine shows the overall exact match rate, which is the percentage of variants with an annotation from either ANNOVAR or VEP (‘ANV+VEP’) that have an exactly matching annotation from the two tools. Here, the specific annotations from equivalent terms for ANNOVAR and VEP have been aggregated to enable the comparison (see Additional file 1: Table S4). The final three rows of the table show aggregate counts and match rates for all loss-of-function categories, all LoF and missense categories and all exonic categories, respectively. Note that the all splicing category for VEP comprises 5,011 splice acceptor variants, 8,544 splice donor variants and 49,298 more general splice region variants. ANNOVAR, in contrast, only has one general splicing category, and does not distinguish between acceptor, donor and other splicing variants.

In total, 637,841 variants were given exonic annotations by either ANNOVAR or VEP (Table 2). Of these, 551,983 (86.5%) had exactly matching annotations from the two tools and 556,387 (87.2%) have category matching annotations. However, the match rate is substantially lower (65% for exact matches, 66% for category matches) for LoF annotations (Table 2). We observe that 89% of exonic variants from VEP get an exactly matching annotation from ANNOVAR and 96% of exonic variants according to ANNOVAR get an exactly matching annotation from VEP. These percentages of agreement should not be taken to show that ANNOVAR is ‘more accurate’ than VEP – the difference between the tools for exonic variants is driven by the larger number of splicing annotations from VEP, which is due to a difference in the definition of a splicing variant used by the two tools.

Considering all annotation categories for VEP and ANNOVAR annotations shows a substantial amount of disagreement in annotations from the two tools, even when using the same transcripts (Additional file 1: Figures S1 and S2). We observe relatively lower concordance for intergenic, intronic, miRNA and splicing variants. Even in well-defined categories such as nonsynonymous (missense) and frameshift, we see a large amount of disagreement in annotations between the two tools. We saw no significant differences in annotation agreement rates across different variant frequencies (Additional file 1: Table S5b).

To characterise the sorts of apparent errors or inconsistencies that commonly emerge in annotation by ANNOVAR and VEP, we investigated cases for which annotations from ANNOVAR and VEP disagree. Although it is counter-intuitive (since the annotations were based on the same set of transcripts), ANNOVAR and VEP do not always use the same transcript for the annotation of a variant. This is a result of the interaction of different annotation categories, different precedence rules and the fact (for this study) of reporting only one consequence for each variant. When characterising differences and apparent errors in annotation, we looked at variants for which we know ANNOVAR and VEP did indeed use the same transcript as the basis for annotation. We focused on LoF variants – frameshift, stop-gain, stop-loss and splicing – as they are currently of most interest in disease studies, and we saw better than 90% agreement between ANNOVAR and VEP annotations for nonsynonymous and synonymous variant categories (Table 2). Where possible (as in the case of splicing annotations), we discuss differences in annotation algorithms that are likely causes of differences in annotation, but detailed information on annotation algorithms is not available for ANNOVAR or VEP, even in online documentation [49,50].

#### Frameshift variants

We observed over 2,000 variants that are annotated as frameshift by either ANNOVAR or VEP but not the other (Additional file 1: Table S6). Among these, we found that ANNOVAR annotates over 300 variants as frameshift despite them being SNVs, so the ANNOVAR annotation is unequivocally incorrect for these variants. For the majority of these variants, however, it is not possible to say conclusively from manual inspection whether the ANNOVAR or VEP annotation is correct.

All of the variants that are annotated as frameshift by VEP but not by ANNOVAR are genuine indels and none are a multiple of three bases, so VEP looks to be correctly identifying these variants as frameshift indels. Several hundred variants get nonframeshift, nonsynonymous and synonymous annotations from ANNOVAR, which are incompatible with the frameshift annotations from VEP. The frameshift annotations seem reasonable, so ANNOVAR looks to give incorrect annotations for these variants. Several hundred other variants are annotated as stop-gain by ANNOVAR and frameshift by VEP. A stop-gain annotation is not necessarily incompatible with a frameshift annotation from VEP, as ANNOVAR inspects the transcript produced by the insertion/deletion and sometimes finds that a stop codon is introduced by the indel. Following its precedence rules, it then returns an annotation of stop-gain rather than frameshift. The disagreement between annotations for such variants is thus reasonable once we take into account how the two tools report annotations. From looking at specific examples it appears that only a small fraction of variants get an incorrect annotation from both software tools.

#### Stop-gain variants

When we look at the variants annotated as stop-gain by ANNOVAR, but not by VEP (when the same transcripts are used), we see that the majority (437 of the 570) are given frameshift annotations by VEP (Additional file 1: Table S7). We saw above that ANNOVAR’s precedence rules can lead it to give a stop-gain annotation to an indel for which frameshift would otherwise be a reasonable annotation. Here too, all of the variants annotated as frameshift by VEP seem to be genuine frameshift variants (as they are indels that are not a multiple of 3 bp in size). These discrepancies, therefore, reflect a difference in precedence for reporting annotations, rather than a true difference between the annotation algorithms, and the ANNOVAR annotation (assuming it correctly identifies introduced stop codons) adds information of interest. There is a much smaller number of variants given missense (77) and synonymous (39) annotations by VEP (Additional file 1: Table S7a).

Manual inspection in the ENSEMBL Genome Browser of ten of those discrepant variants on chromosome 1 shows that for eight of the ten missense (from VEP) variants, the VEP annotation looks correct (for two variants neither annotation looks correct; see Additional file 1: Table S10 for details of these variants). For other discrepant variants, manual inspection reveals that the VEP annotation looks correct more often than the ANNOVAR annotation (see Additional file 1: ‘Supplementary Results’ for more details). When we look at variants annotated as stop-gain by VEP and either frameshift or nonframeshift by ANNOVAR, we see that approximately 20% (30 variants) of these are SNVs, which cannot be correctly annotated as frameshift or nonframeshift (as these terms only apply to an insertion or deletion). Thus, the ANNOVAR annotations for these particular variants cannot be correct, and must simply be a result of a software bug. For the remaining variants it is difficult to assess whether the ANNOVAR or VEP annotation is better. Even after taking into account the differences in annotation caused by different precedence rules, the stop-gain annotations from VEP look more reliable than those from ANNOVAR.

#### Stop-loss variants

There are only small numbers of variants that are annotated as stop-loss by ANNOVAR and not by VEP, but almost all of these are annotated as frameshift by VEP. Inspection reveals that all of these variants are indeed indels that are not a multiple of three bases, therefore annotations of frameshift from VEP are reasonable. Looking closely at the variants reveals that there is a roughly even split between when the ANNOVAR or the VEP annotation look better. There are only 16 variants that are annotated as stop-loss by VEP and as something else by ANNOVAR when the two tools use the same transcript for annotation (Additional file 1: Table S8).

#### Splicing variants

The category (or categories) of splicing variants is a source of many differences in annotations from different annotation software tools. Unlike most other categories of annotation, in the field there are still multiple notions of what entails a splicing variant. ANNOVAR defines just one broad category, splicing, for these variants: any variant within *x* bp of a splicing junction receives the annotation splicing. The value of *x* can be specified by the user of ANNOVAR, and for our annotations here we used a broad definition of splicing by setting *x*=6. In contrast, VEP uses three categories of splicing variant: (1) splice donor variant, a splice variant that changes the two-base region at the 5^′^ end of an intron; (2) splice acceptor variant, a splice variant that changes the two-base region at the 3^′^ end of an intron and (3) splice region variant, a sequence variant in which a change has occurred within the region of the splice site, either within one to three bases of the exon or three to eight bases of the intron. VEP thus gives more useful information, through its subcategories of splicing variants, about the likely function of a variant. We also see that differences in annotation can arise simply as a result of differing definitions of what a splicing variant is, rather than any truly substantial differences in the algorithms producing the annotations. We investigated these differences in annotation on variants where both tools used the same transcript for annotation, and annotations did not match, that is, a variant with a splicing annotation from ANNOVAR did not get an annotation of one of splice donor variant, splice acceptor variant or splice region variant, or the inverse.

The major source of difference in splicing annotations is that the overwhelming proportion of ANNOVAR splicing variants that receive non-splicing annotations from VEP actually receive one of VEP’s three splicing annotations, but reported as being in a non-coding transcript (Additional file 1: Table S9). This result suggests that VEP does a better job at reporting when the transcript it uses for annotation is non-coding, but that there may actually not be such a large degree of difference between splicing annotations as appears initially. We also see here the combined effect of different definitions of splicing variants and precedence rules that result in a splicing variant found in one transcript being reported instead of a less ‘serious’ variant seen in another transcript. We see a large number of variants annotated as synonymous by ANNOVAR and as splice region variant by VEP, and all are in an exon, either in the first three bases (5^′^ end) or last three bases (3^′^ end) of the exon. Thus, these annotation differences seem to be a systematic result of differences in the annotation algorithms used by ANNOVAR and VEP, and for these variants the VEP annotations look to be better.

### Discussion

The results of our comparison of annotations obtained using REFSEQ and ENSEMBL transcript sets emphasise the importance of the choice of transcript set used for annotation. Applying the same annotation software with different transcript sets saw a matching rate of 44% for putative LoF annotations. Though not done here, transcript sets from REFSEQ and ENSEMBL (or other sources) can be restricted to a subset of transcripts to exclude low confidence annotations. Where a specific tissue of interest is known, annotation could be restricted to use only the set of transcripts known to be expressed in that tissue. Defining a targeted set of transcripts will not always be easy, but for sequencing studies where the cost of false positives (e.g. through follow-up experiments) is high, and where information on the expression of specific transcripts exists, a set of high-confidence transcripts tailored to the study at hand may be preferable. Projects like GENCODE aim to provide a carefully curated transcript set supported by experimental evidence [15,51-53], so through efforts such as these we may see annotation results converge as (ideally tissue-specific) transcript sets align across different repositories. For the time being, though, large differences remain.

Variant annotation remains challenging for current software tools: differing choices made in annotation packages on how to analyse, categorise and prioritise annotations for a variant lead to differing annotations from different tools, even when using the same set of transcripts as the basis for annotation. Differences in annotations from different software tools (e.g. 64% overall agreement for LoF annotations) are not as large as those seen when using different transcript sets (44% overall agreement for LoF annotations), and are often caused by differences in the annotation categories defined by different tools. Nevertheless, the extent of the differences seen show that, again, careful consideration must be given when choosing a software tool to make sure that it is well suited to the goals of the scientific investigation.

Standardising definitions of variants across the field, to reduce the scope for apparent differences in annotations returned by different software tools and to crystallise the (epistemic) meaning of terms used for annotations, could be of value. In our results here, for example, differing definitions of splicing variants cause tens of thousands of annotation differences. The Sequence Ontology Project [54] may help with this. It would be beneficial for phase information to be used in annotating variants in close proximity, given, for example, the extent of ‘rescue’ of LoF variants by nearby variants [55]. Currently, annotation tools typically do not associate any measure of uncertainty with reported variant annotations. Such information could be useful for downstream analysis, especially for consideration when allocating resources for follow-up experiments on variants of interest. When a high level of certainty about the validity of an annotation is required, variants could be annotated with two software tools and variants with differing annotations flagged to be treated with caution.

In the comparison of annotation tools here, we restricted each tool to report just the most severe consequence annotation for each variant, to avoid comparisons becoming too unwieldy. However, VEP and other annotation tools can (and often by default do) report annotations for all transcripts, providing extra information that is often valuable. Adding this extra information, as with utilising phase information or tissue-specific transcripts, increases the challenges for data processing and interpretation by adding complexity to the treatment of variant annotation, but with good reason: this added complexity reflects the underlying biology, so taking this information into account potentially adds significant value to analyses of DNA variants.

Our understanding of the human genome continues to improve rapidly even as we gain a better appreciation of the genome’s complexity. As a result, at some point we may see the variant annotations from different approaches converge. For the time being, though, we confront an epistemic challenge (determining the meaning or function of variants observed) because our ontological foundations (knowledge and understanding of what all sequences in the genome actually do) remain unresolved or unclear. Thus, the choices of transcript set and software tool can have substantial effects on the annotation results obtained, and from there, large effects on all downstream aspects of the analysis of WGS data. Variant annotation is not yet a plug-and-play procedure and should not be treated as such.

In addition to different variant annotation approaches (of which there are more than we have compared here), there are different sequencing technologies, read mappers and variant callers. Each of these can potentially have substantial impact on the final variants and annotations obtained, but comparison of other sources of variation is beyond the scope of this paper. We refer interested readers to systematic comparisons of other aspects of the next-generation sequencing pipeline, for example comparisons of benchtop high-throughput sequencing technologies [56], short-read mappers [57], variant callers [58] and variant-calling pipelines as a whole [59,60].

We have aimed to highlight the effect on final annotation results that can arise from two aspects of analyses of whole genome (or whole exome) sequence data, namely, choice of transcript and choice of annotation software. While we are not advocating any particular software or transcript set, we suggest researchers be aware of the impact of these choices, and hope our comparisons may inform such decisions.

## Conclusions

We have quantified the extent of disparity in variant annotation when different transcript sets and different software tools are used. This comparison of annotations for 80 million human DNA variants revealed many substantial differences between annotations based on different transcript sets and different software tools. The extent of differences in annotations was particularly large in annotation categories of most interest, namely, putative LoF and nonsynonymous variants. We found many more variants with annotations in interesting categories when using ENSEMBL transcripts compared with REFSEQ transcripts only. If it is important not to miss potential LoF variants, then there are advantages to using ENSEMBL transcripts. If it is important to reduce false positives, then a carefully curated set of transcripts tailored to the study at hand may be preferred. Even when using the same transcript set, different annotation software packages can provide substantially different annotations.

There are variants with potentially severe effects that are identified with one method and not another. We require consistent, accurate and reliable annotation of variants to support the use of WGS in making diagnostic and treatment decisions. The dependence of current annotation results on the set of transcripts and software used can be managed, with sufficient care, in the research context. However, more work is required to improve variant annotation for clinical use. The differences in annotation due to choice of transcript set and software package quantified here should be given due consideration when undertaking variant annotation in practice. Careful thought needs to be given to the choice of transcript sets and software packages for variant annotation in sequencing studies.

## Abbreviations

bp: base pair; CCDS: Consensus Coding Sequence; LoF: loss of function; Mb: megabase; miRNA: microRNA; SNV: single nucleotide variant; UTR: untranslated region; VEP: Variant Effect Predictor; WGS: whole-genome sequencing.

## Competing interests

The WGS500 Project was funded in part by Illumina, Inc.

## Authors’ contributions

DJM conducted the analysis and wrote the manuscript. PH, MAR and KG wrote alternative software for variant annotation, which was used to compare with Annovar, and provided advice about the comparisons. AK processed raw sequencing data, called variants and conducted the annotation using Annovar. JBC supervised the analysis and wrote the manuscript. PD conceived of the study, directed the process and wrote the manuscript. The WGS500 Consortium provided the raw data. A list of WGS500 Consortium members and affiliations is provided in Additional file 1. All authors approved the final version of the manuscript.

## Supplementary Material

Additional file 1 **Supplementary material for ‘Choice of transcripts and software has a large effect on variant annotation’.** This PDF file contains supplementary figures, supplementary tables and further details of the results of the annotation comparisons for which there was insufficient space in the main text. Click here for file
