# Supplementary material for: Choice of transcripts and software has a large effect on variant annotation
Source: Genome Med. 2014 Mar 31;6(3):26. doi: 10.1186/gm543 (PMC4062061; doi:10.1186/gm543)
Supplement: Additional file 1 — Supplementary material for ‘Choice of transcripts and software has a large effect on variant annotation’. This PDF file contains supplementary figures, supplementary tables and further details of the results of the annotation comparisons for which there was insufficient space in the main text. [file gm543-S1.pdf]

Supplementary Material for: "Choice of transcripts and software has a large effect on variant annotation"

Davis J McCarthy*[1,2], Peter Humburg[2], Alexander Kanapin[2], Manuel A Rivas[2], Kyle Gaulton[2], WGS500 Consortium, Jean-Baptiste Cazier[3]and Peter Donnelly[1,2]

[1]Department of Statistics, University of Oxford, South Parks Road, Oxford, UK
[2]Wellcome Trust Centre for Human Genetics, University of Oxford, Roosevelt Drive, Oxford, UK
[3]Department of Oncology, University of Oxford, Roosevelt Drive, Oxford, UK

Email: Davis J McCarthy*- davis.mccarthy@well.ox.ac.uk; Peter Donnelly - donnelly@well.ox.ac.uk;

*Corresponding author

Abstract

This document contains Supplementary Material for "Choice of transcripts and software has a large effect on variant annotation". It includes details of the WGS500 consortium, more discussion of differences between RefSeq and Ensembl, more detailed discussion of results that could not be included in the main text, supplementary figures providing conrete examples of annotation differences and supplementary tables presenting extra results for the comparison of annotation methods.

Contents

1 500 Whole-Genomes (WGS500) Consortium Membership 3

2 More information on RefSeq and Ensembl transcript sets 4

3 Supplementary Results 5

4 Supplementary Figures 10

5 Supplementary Tables **20**

List of Figures

1 Annovar-normalized heatmap . . . 11
2 VEP-normalized heatmap . . . 12
3 Browser Image: RefSeq synonymous, Ensembl stoploss . . . 13
4 Browser Image: RefSeq stoploss, Ensembl synonymous . . . 14
5 Browser Image: RefSeq synonymous, Ensembl stopgain . . . 14
6 Browser Image: RefSeq stopgain, Ensembl synonymous . . . 15
7 Browser Image: RefSeq intronic, Ensembl frameshift deletion . . . 16
8 Browser Image: RefSeq frameshift insertion, Ensembl frameshift deletion . . . 17
9 Browser Image: RefSeq nonsynonymous, Ensembl splicing . . . 18
10 Browser Image: RefSeq splicing, Ensembl synonymous . . . 19

List of Tables

1 RefSeq/Ensembl full comparison . . . 21
2 Annovar/VEP full comparison . . . 22
3 VEP precedence values . . . 23
4 Annovar and VEP terms . . . 24
5 Matching rates across MAF ranges . . . 24
6 Annotation differences: frameshift by only one of Annovar or VEP . . . 25
7 Annotation differences: stop-gain by only one of Annovar or VEP . . . 26
8 Annotation differences: stop-loss by only one of Annovar or VEP . . . 27
9 Annotation differences: splicing by only one of Annovar or VEP . . . 28
10 Annovar “stop-gain”, VEP “missense” variants . . . 29

1 500 Whole-Genomes (WGS500) Consortium Membership

Steering Committee

Peter Donnelly[1] (Chair), John Bell[2] , David Bentley[3], Gil McVean[1], Peter Ratcliffe[1], Jenny Taylor[1,4], Andrew Wilkie[4,5]

Operations Committee

Peter Donnelly[1] (Chair), John Broxholme[1], David Buck[1], Jean-Baptiste Cazier[1], Richard Cornall[1], Lorna Gregory[1], Julian Knight[1], Gerton Lunter[1], Gilean McVean[1], Jenny Taylor[1,4], Ian Tomlinson[1,4], Andrew Wilkie[4,5]

Sequencing & Experimental Follow up

David Buck[1] (Lead), Christopher Allan[1], Moustafa Attar[1], Angie Green[1], Lorna Gregory[1], Sean Humphray[3], Zoya Kingsbury[3], Sarah Lamble[1], Lorne Lonie[1], Alistair Pagnamenta[1,4], Paolo Piazza[1], Guadelupe Polanco[1], Amy Trebes[1]

Data Analysis

Gil McVean[1] (Lead), Peter Donnelly[1], Jean-Baptiste Cazier[1], John Broxholme[1], Richard Copley[1], Simon Fiddy[1], Russell Grocock[3], Edouard Hatton[1], Chris Holmes[1], Linda Hughes[1], Peter Humburg[1], Alexander Kanapin[1], Stefano Lise[1], Gerton Lunter[1], Hilary Martin[1], Davis McCarthy[1], Lisa Murray[3], Andy Rimmer[1], Natasha Sahgal[1], Ben Wright[1], Chris Yau[6]

[1] *The Wellcome Trust Centre for Human Genetics, Roosevelt Drive, Oxford, OX3 7BN, UK.*

[2] *Office of the Regius Professor of Medicine, Richard Doll Building, Roosevelt Drive, Oxford, OX3 7LF, UK.*

[3] *Illumina Cambridge Ltd., Chesterford Research Park, Little Chesterford, Essex, CB10 1XL, UK.*

[4] *NIHR Oxford Biomedical Research Centre, Oxford, UK.*

[5] *Weatherall Inst of Molecular Medicine, University of Oxford; John Radcliffe Hospital Headington, Oxford OX3 9DS, UK.*

[6] *Imperial College London, South Kensington Campus, London, SW7 2AZ, UK.*

2 More information on RefSeq and Ensembl transcript sets

The results in the main manuscript show a large effect on functional annotations due to choice of transcript set. This effect arises because transcript sets from different sources can have different characteristics. Both Ensembl and RefSeq contain transcripts established from experimental evidence. RefSeq transcripts are constructed from sequence data submitted to the International Nucleotide Sequence Database Collaboration (INSDC) [1]. Similarly, all Ensembl transcripts are based on experimental evidence, namely mRNAs and protein sequences deposited in public databases [2]. Both transcript sets contain transcripts produced by automated pipelines run on the database sequences and manually-curated transcripts.

A portion of the RefSeq dataset is manually curated by National Center for Biotechnology Information (NCBI) staff, with the remainder produced from the automated annotation pipeline. External information is incorporated entirely into RefSeq's organizing framework. In contrast, Ensembl more explicitly imports information from external sources:

> ... transcripts from the Consensus Coding Sequence (CCDS) set are imported directly and not altered by the genebuild process. In addition, where manual curation is available for a transcript, the Ensembl and HAVANA transcript models are compared. The Ensembl and HAVANA models are merged when they agree on the same coding sequence. Merged models will be coloured gold in the browser. A merged, or golden, gene indicates one or more common transcripts between Ensembl and HAVANA. This combined geneset is the default gene set from the GENCODE project [2].

Although these approaches from RefSeq and Ensembl are broadly similar, the resulting transcript sets are substantially different.

Unfortunately, detailed information about the automated pipelines and manual curation processes is not generally available. The quality assessment processes and inclusion requirements for both transcript sets are necessarily extensive and complicated and the transcript sets change regularly according to planned release schedules. As a result, detailed information on the breakdown of levels of support (evidence) for the human transcripts included in each set (e.g. proportion of transcripts from an automated pipeline versus the proportion that are manually curated) is not available, preventing us from a thorough characterisation

of why certain transcripts may appear in the RefSeq set and not the Ensembl set and vice versa.

As noted in the main text, the Ensembl transcript set contains many more transcripts than RefSeq and the RefSeq transcript set is not simply a subset of the Ensembl set. The RefSeq frequently asked questions website [3] offers some insight:

> RefSeq records that represent alternately spliced transcript variants are provided when there is experimental and/or published evidence in support of the full-length nature of the product. When transcript alignments (to the assembled genome) indicate that there is alternate splicing no assumption is made about the naturally-occurring combination(s) of alternate exons in the absence of full-length support. As a consequence, alternately spliced products are underrepresented in the RefSeq collection.

The difference in the treatment of alternately spliced products is likely to contribute to the substantially greater number of transcripts in the Ensembl set than in the RefSeq set.

More information on the RefSeq and Ensembl datasets is available from the NCBI Handbook [1], and the RefSeq and Ensembl websites [4, 5].

3 Supplementary Results

Here we provide more details on the results of the annotation comparison between Annovar and VEP with Ensembl transcripts than was possible to include in the main text.

The heatmaps in Supplementary Figures 1 & 2 represent the normalized counts for each combination of VEP and Annovar annotation. The Annovar- and VEP-normalized counts were computed in an analogous fashion to the computation of the RefSeq- and VEP-normalized counts in the transcript set comparison above. For an annotation term category under consideration for one software tool, counts across all categories from the other software tool are mean-centered and divided by the standard deviation, giving normalized counts that indicate, for a given annotation term for a given software tool, the relative breakdown of annotations from the other software tool. Ideally, we would like to see, for example, that all variants called “synonymous” by VEP are also annotated as “synonymous” by Annovar and vice-versa. Similarly, we would like to see agreement in annotations across all categories. Annovar-normalized values (Supplementary Figure 1) indicate generally good agreement of Annovar annotations with VEP

annotations. Nevertheless, there are substantial numbers of variants receiving differing annotations from the two tools across all categories of variants. The VEP-normalized values (Supplementary Figure 2) confirm this view. We observe lower concordance for intergenic, intronic, miRNA and splicing variants.

To characterise the sorts of apparent errors or inconsistencies that commonly emerge in annotation by Annovar and VEP, we need to investigate specific examples for which the Annovar or VEP annotation appears dubious by looking closely at variants for which annotations from Annovar and VEP disagree.

For the four LoF categories—frameshift, stop-gain, stop-loss and splicing—we look at the numbers of variants that are given that annotation by Annovar and not VEP, and vice versa. We look at which categories most frequently crop up in the "disagreeing" annotations. Further, we examine (in the Ensembl Web Browser; browser results not shown) some specific examples of variants with disagreeing annotations to try to characterise the causes of some of the differences in annotations from Annovar and VEP. We focus our attention particularly on cases where the two software tools used the same transcript for information, as these cases are better able to give us insight into the different characteristics of the differing annotations.

Frameshift variants

Annovar annotates 15,822 variants as "frameshift", of which 13,486 (85%) also receive a "frameshift" annotation from VEP (Table 2 in the main text). This leaves 2,336 variants annotated as "frameshift" by Annovar that get a different annotation from VEP. Of these, both tools used the same transcript for 1,333 variants and they used different transcripts for the remaining 1,003 variants.

When matching transcripts were used, 905 of the 2,015 variants were annotated as "missense" by VEP, and 257 as "synonymous" (the two most common categories; Supplementary Table 6a). Remarkably, 54% of these variants are single nucleotide variants (data not shown), which indicates that the Annovar algorithm is making errors in these cases.

There are some cases where neither tool seems to get the annotation correct. For example, the variant 1:52499090_G_GGGTTCT is a 6bp insertion, so it seems that "inframe insertion" would be the best annotation, but it is annotated as "frameshift" by Annovar and "missense" by VEP. From looking at specific examples it appears that only a tiny fraction of variants get an incorrect annotation from both

software tools.

VEP annotates 16,685 variants as "frameshift", of which 13,486 (81%) also receive a "frameshift" annotation from Annovar (Table 2). This leaves 3,199 variants annotated as "frameshift" by VEP that get a different annotation from Annovar. The two tools used the same transcript for 1,300 of these variants and different transcripts for the remaining 1,899 variants (Supplementary Table 6b. All of the variants annotated as "frameshift" by VEP are indels (single nucleotide variants) and none are a multiple of three bases, so VEP looks to be correctly identifying frameshift indels.

When matching transcripts are used, 394 variants of the 1,300 variants are annotated as "nonframeshift" by Annovar, 437 as "stopgain" and 283 as "nonsynonymous" (Supplementary Table 6b). The "nonframeshift" and "nonsynonymous" annotations from Annovar are incompatible with the "frameshift" annotations from VEP and the "frameshift" annotations seem reasonable, so Annovar seems to give incorrect annotations for these variants. The variants annotated as "stopgain" by Annovar are interesting, as this category is not necessarily incompatible with a "frameshift" annotation from VEP. Indeed, these are frameshift indels, but Annovar looks closely at the transcript produced by the insertion/deletion and sometimes finds a stop codon is introduced. Following its precedence rules, it then returns an annotation of "stopgain" rather than "frameshift". The disagreement between annotations for such variants is thus reasonable once we take into account how the two tools report annotations.

Stop-gain variants

Annovar annotates 14,960 variants as "stopgain" and 96% of these are annotated as "stop-gain" by VEP too (Table 2). This leaves 612 variants that get different annotations from VEP. The two tools use the same transcript to annotate 570 of this variants, and different transcripts for the remaining 42 variants.

When matching transcripts are used, 437 of the 570 variants with discrepant annotations are given "frameshift" annotations by VEP. We saw above that the Annovar's precedence rules can lead it to give a "stopgain" annotation to an indel for which "frameshift" would otherwise be a reasonable annotation. Here too, all of the variants annotated as "frameshift" by VEP seem to be genuine frameshift variants, so the Annovar annotation (assuming it correctly identifies introduced stop codons) adds some more information of interest. There is a much smaller number of variants given "missense" (77) and "synonymous" (39) annotations by VEP (Supplementary Table 7a). Looking up the those variants on

chromosome 1 shows that for 8 of the 10 "missense" (from VEP) variants, the VEP annotation looks correct (for two variants neither annotation looks correct). Of the two given "synonymous" annotations by VEP, the ANNOVAR annotation looks correct for one and the VEP annotation looks correct for the other.

VEP annotates 16,146 variants as "stop-gain" and 89% of these are also annotated as "stopgain" by ANNOVAR (Table 2). Thus, 1,798 variants get different annotations from ANNOVAR, of which the two tools used the same transcript for 225 variants and different transcripts for 1,573 variants (with 91% of those getting an "intergenic" or "unknown" annotation from ANNOVAR; Supplementary Table 7b).

When matching transcripts are used, the breakdown of the ANNOVAR annotations for the 225 variants is 74 "frameshift", 52 "nonframeshift", 77 "nonsynonymous" and 22 "synonymous" (Supplementary Table 7b). 30 of the "frameshift" or "nonframeshift" (from ANNOVAR) variants are single nucleotide variants, so an annotation of "frameshift" or "nonframeshift" cannot be correct. For the remaining genuine indels, VEP finds a stop codon introduced where ANNOVAR does not—these variants are difficult to eyeball to see which annotation is correct. The "frameshift"/"nonframeshift" annotations from ANNOVAR are reasonable, but if the VEP annotations are correct then annotating the variants as "stop-gain" provides more useful information. Checking the 8 variants on chromosome 1 given "nonsynonymous" or "synonymous" annotations by ANNOVAR indicates that the VEP annotation is correct for 5 variants, and for the other 3 the correct annotation was not possible to determine by eyeballing the variant in the browser. Overall, the "stop-gain" annotations from VEP look substantially more reliable than those from ANNOVAR.

Stop-loss variants

ANNOVAR annotates 906 variants as "stop-loss" of which 870 are also annotated as "stop-loss" by VEP (Table 2). Therefore, only 36 variants get different annotations from VEP, 30 of which were annotated with the same transcript by both tools, and 6 of which were annotated with different transcripts.

When matching transcripts are used, 25 of the 30 variants are given "frameshift" annotations by VEP (Supplementary Table 8a). These "frameshift" variants are all indeed indels that are not a multiple of three bases—as such, annotations of "frameshift" from VEP are reasonable. Across a selection of 22 of the 30 variants, it seems that ANNOVAR gives the best annotation for 8 variants, VEP gives the best annotation

for 11 variants and neither appears to give the best possible annotation for the remaining 3 variants.

VEP annotates 1,077 variants as "stop-loss", of which 870 (81%) are also annotated as "stop-loss" by ANNOVAR (Table 2). That leaves 207 variants that are given different anntoations by ANNOVAR. The two tools use the same transcript for only 16 of these variants, using different transcripts for the other 191 (Supplementary Table 8b). When matching transcripts are used we therefore see a negligibly small number of variants that are not given a "stop-loss" annotation by ANNOVAR when they are annotated as "stop-loss" by VEP. These results support the notion that VEP is doing an excellent job of annotating "stop-loss" variants.

Splicing variants

ANNOVAR annotates 45,839 variants as "splicing", of which 86% receive a splicing annotation from VEP (Table 2). Of those 6,259 variants with differing annotations from VEP, the two tools used the same transcript for 119 variants and different transcripts for the remaining 6,140 variants. The major source of difference in "splicing" annotations is that the overwhelming proportion of ANNOVAR "splicing" variants that receive non-"splicing" annotations from VEP actually receive one of VEP's three splicing annotations, but reported as being in a non-coding transcript (Supplementary Table 9a). This suggests that VEP does a better job at reporting when the transcript it uses for annotation is non-coding, but that there may actually not be such a large degree of difference between splicing annotations as appears initially. We also see here the combined effect of different definitions of splicing variants and precedence rules that result in a splicing variant found in one transcript being reported instead of a less "serious" variant seen in another transcript.

VEP annotates 62,853 variants in one of its three "splicing" categories, of which 63% are also annotated as "splicing" by ANNOVAR. Thus, there are 23,273 variants that are annotated as "splicing" by VEP and not by ANNOVAR. Of these, the two tools used the same transcript for 3,540 variants (15%) and different transcripts for the other 19,733 variants.

When matching transcripts are used, 88% of the 3,540 variants are given "synonymous" annotations by ANNOVAR (Supplementary Table 9b). Looking closely in the ENSEMBL Web Browser at 20 of these "synonymous" (according to ANNOVAR) variants reveals them all to be annotated as "splice region variant" by VEP, and all are in an exon, either in the first 3 bases (5' end) or last 3 bases (3' end) of the

exon. Thus, these annotation differences seem to be a systematic result of differences in the annotation algorithms used by ANNOVAR and VEP, and for these variants the VEP annotations look to be better.

4 Supplementary Figures

This section contains heatmaps showing the differences in annotations obtained using ANNOVAR and VEP, and a series of Ensembl Web Browser images highlighting variants with different annotations from REFSEQ and ENSEMBL. Details of the variants used for these examples as well as many more examples of variants with different annotations from REFSEQ and ENSEMBL transcripts can be found in the tab-delimited file `rfs_ens_interesting_diffs.tab`, which can be downloaded from the figshare repository at http://dx.doi.org/10.6084/m9.figshare.798828.

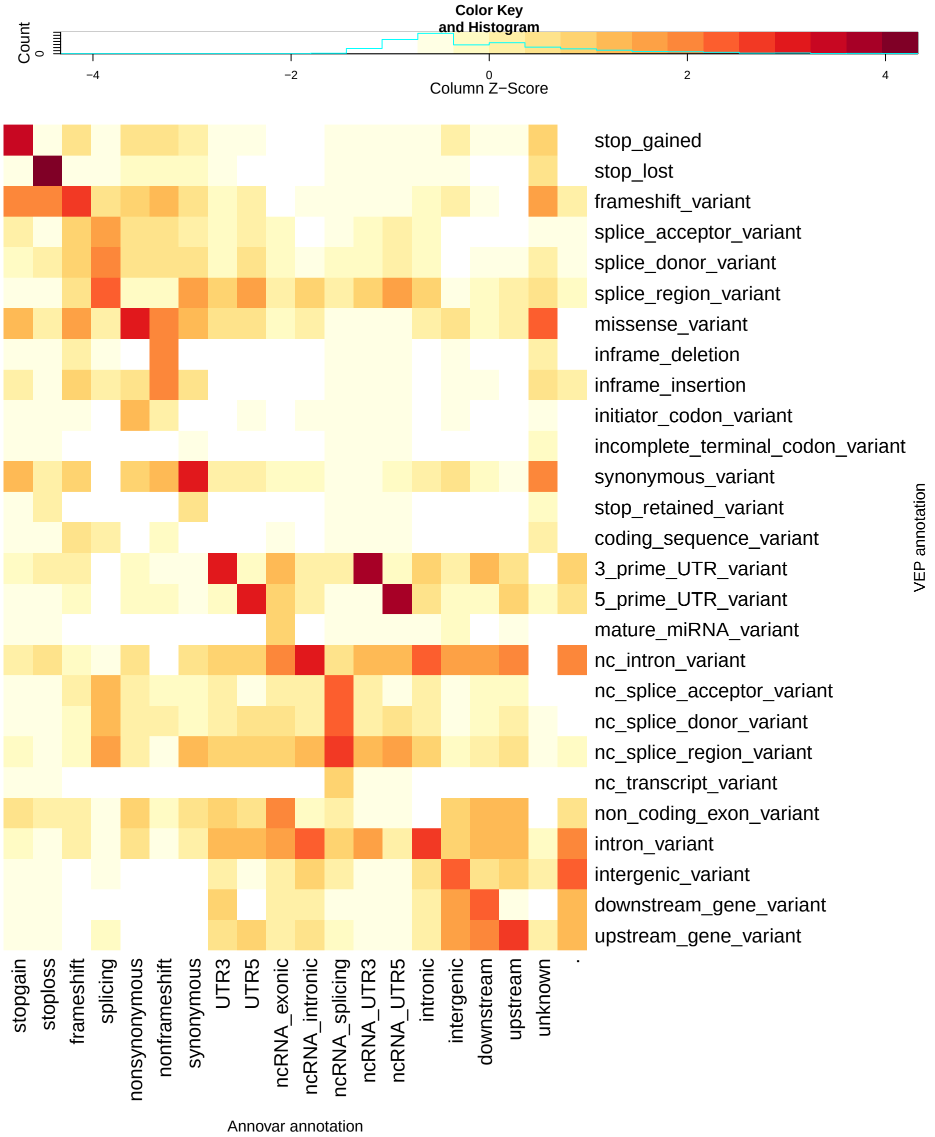

Figure 1: **Annovar-normalized heatmap:** This heatmap shows scaled numbers of variants for all different combinations of categories of annotations when using the VEP annotation tool (rows) and the Annovar software (columns), with the Ensembl transcript set. The values (log10 of the count of variants with that combination of annotations from the two tools, with an offset of 1 applied; see Supplementary Table 2 for raw counts) are Z-scaled (mean-centred, divided by standard deviation) by column (i.e. standardising Annovar annotations). The key above the heatmap shows the values indicated by different colours. Annovar annotation categories are ordered similarly to Table 2, but with all categories of annotation represented in loosely decreasing order of severity, and VEP categories are ordered to correspond (as far as possible) with their matching Annovar categories. This column-normalized heatmap allows us to see which categories of annotation are overrepresented (relative to the total number of variants in the column/category) in the VEP annotations for each category (i.e. column) of Annovar annotation. Contrast this figure with Supplementary Figure 2 and compare with Supplementary Table 2, which provides the counts used for this heatmap.

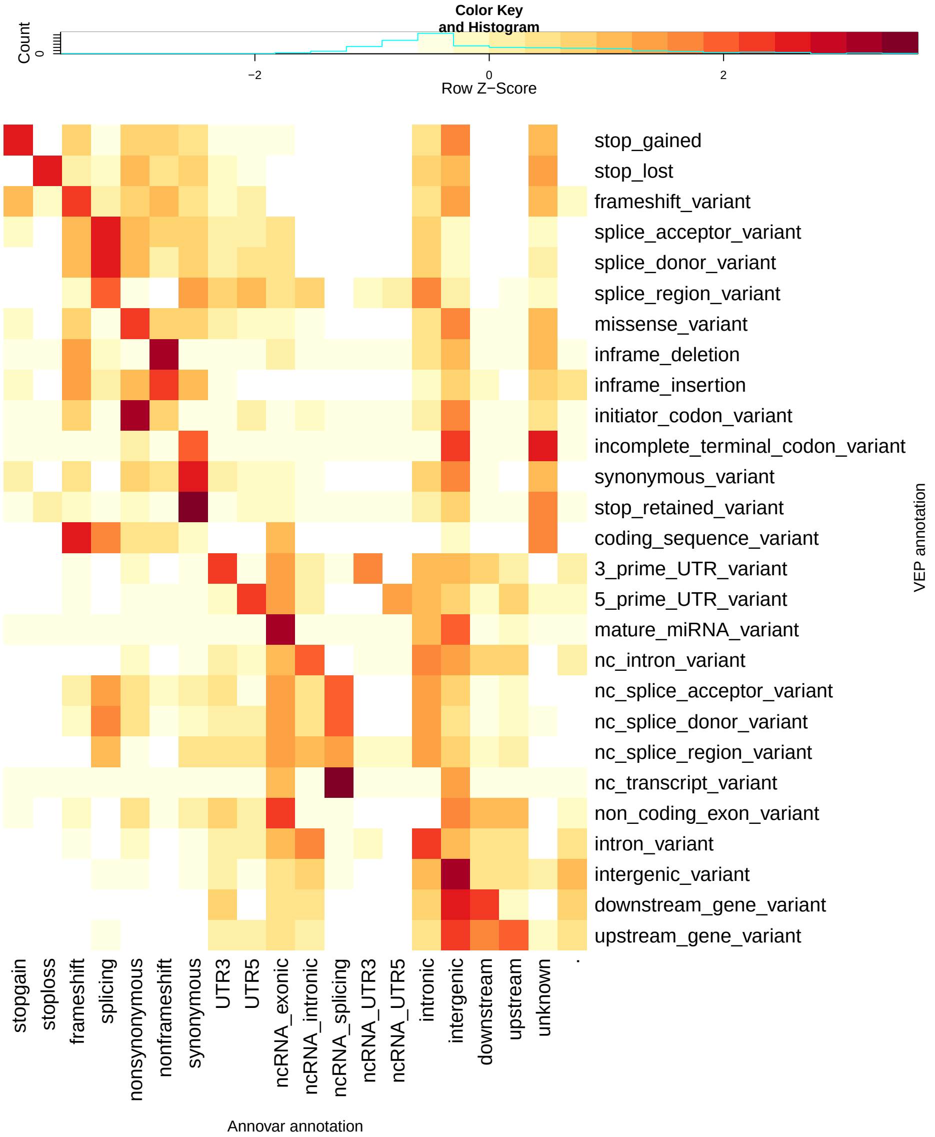

Figure 2: **VEP-normalized heatmap:** This heatmap shows scaled numbers of variants for all different combinations of categories of annotations when using the VEP annotation tool (rows) and the Annovar software (columns), with the Ensembl database. The values (log10 of the count of variants with that combination of annotations from the two tools, with an offset of 1 applied; see Supplementary Table 2 for raw counts) are Z-scaled (mean-centred, divided by standard deviation) by row (i.e. standardising VEP annotations). The key above the heatmap shows the values indicated by different colours. Categories are ordered as per Figure 4. This row-normalized heatmap allows us to see which categories of annotation are overrepresented (relative to the total number of variants in the row/category) in the Annovar annotations for each category (i.e. row) of VEP annotation. Contrast this figure with Figure 4 and compare with Supplementary Table 2, which provides the counts used for this heatmap.

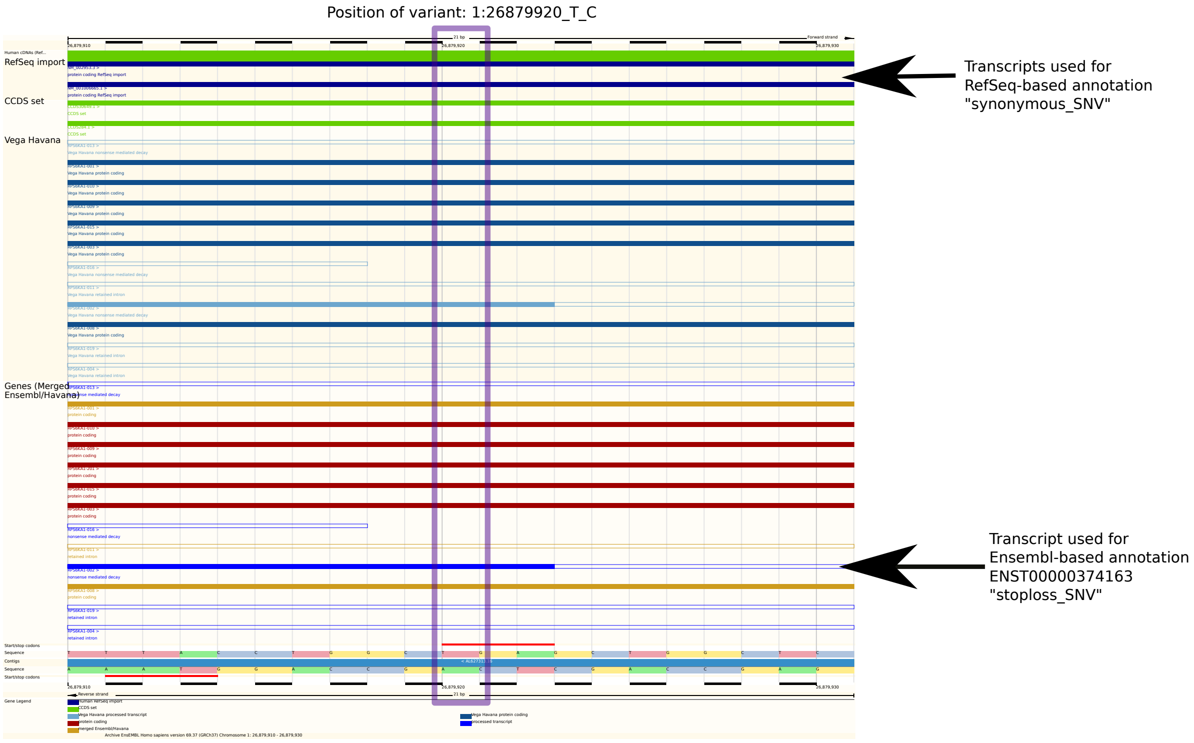

Figure 3: **Browser Image: RefSeq synonymous, Ensembl stoploss.** For this variant, 1:26879920_T_C, the different annotations from RefSeq and Ensembl are due to the use of transcripts with substantially different structures. There are many Ensembl transcripts that would give the same annotation as from the RefSeq transcript, but the one used is the one that gives the most severe consequence, in this case a stoploss variant. Noteworthy is the fact that the Ensembl transcript used is labelled as being subject to nonsense-mediated decay (NMD). So although it is a possible choice of transcript, it may not be the best possible choice, if any changes to this transcript would be irrelevant as the transcript is subject to NDM anyway.

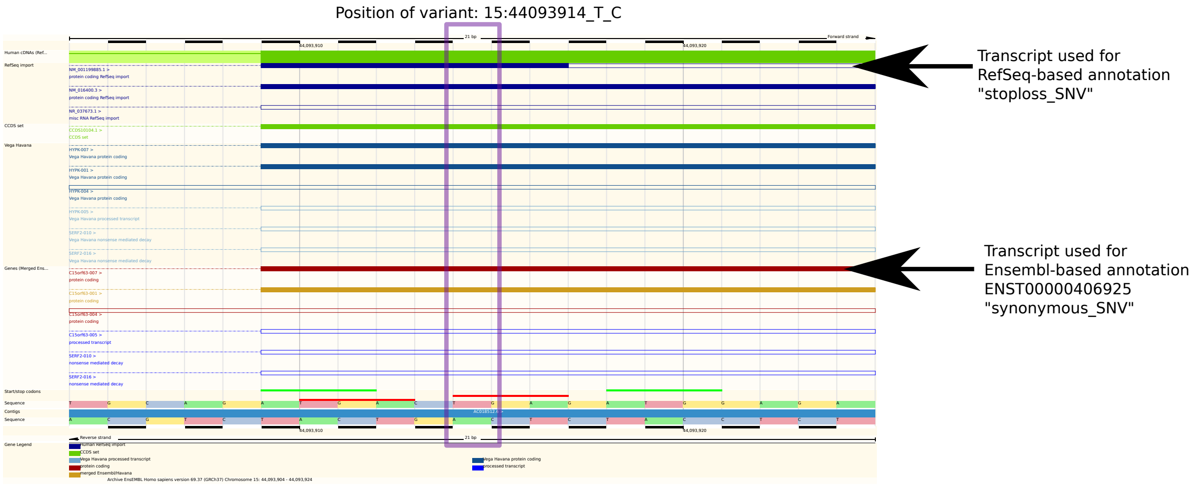

Figure 4: **Browser Image: RefSeq stoploss, Ensembl synonymous.** For this variant, 15:44093914_T_C, the different annotations from RefSeq and Ensembl are due to the use of transcripts with different structures (as seen in Supplementary Figure 3). In this case there are many Ensembl transcripts that look similar to one of the RefSeq transcripts, but one RefSeq transcript is noticeably different from the others. Given the transcripts used, both annotations look "correct" here.

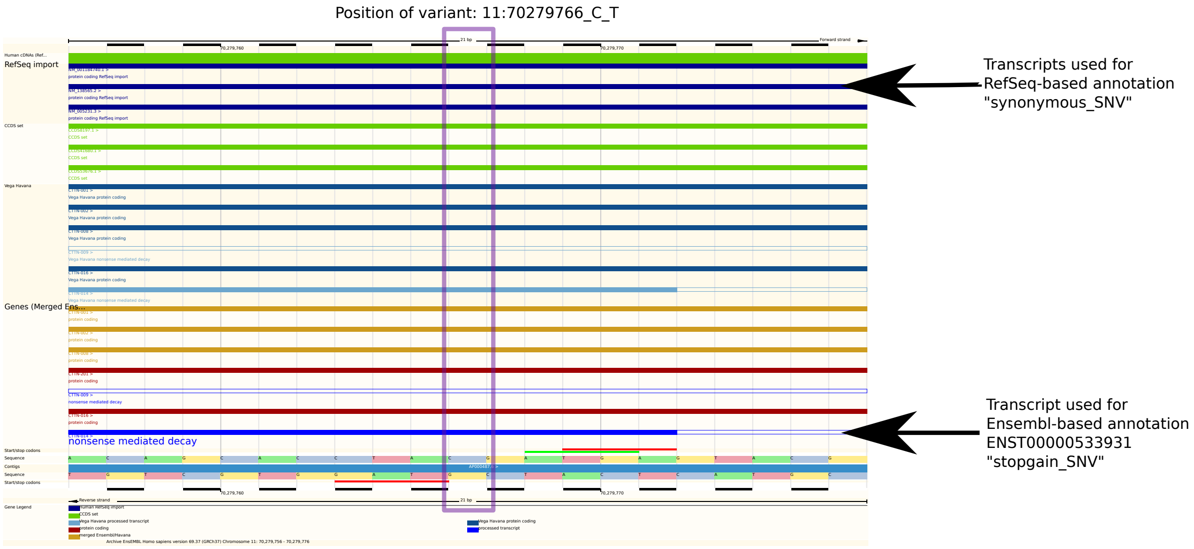

Figure 5: **Browser Image: RefSeq synonymous, Ensembl stopgain.** For this variant, 11:70279766_C_T, the different annotations from RefSeq and Ensembl are again due to the use of transcripts with different structures (as seen in Supplementary Figure 3). In this case there are many Ensembl transcripts that look similar to one of the RefSeq transcripts, but Ensembl used to give the annotation reported is noticeably different from the others, and again noted to be subject to NMD. Given the transcripts used, both annotations look "correct" here, and we note that a difference in the reading frames for the transcripts used accounts for the variant being a "synonymous" variant for many of the transcripts shown and "stopgain" for the Ensembl transcript used.

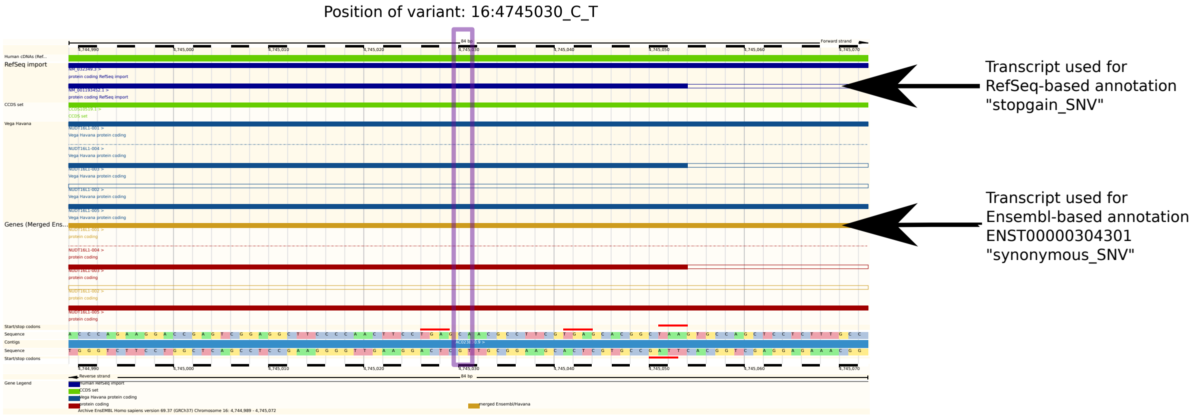

Figure 6: **Browser Image: RefSeq stopgain, Ensembl synonymous.** For this variant, 16:4745030_C_T, the different annotations from RefSeq and Ensembl are again due to the use of transcripts with different structures (as seen in Supplementary Figure 5). Given the transcripts used, both annotations look "correct" here, and again we note that a difference in the reading frames for the transcripts used accounts for the variant being a "synonymous" variant for many of the transcripts shown and "stopgain" for the Ensembl transcript used. What is unclear is why Annovar used the Ensembl transcript that it did, returning a "synonymous" annotation, rather than the shorter red transcript below it, which (it appears) would yield a "stopgain" annotation like the blue RefSeq transcript, which it seems to match.

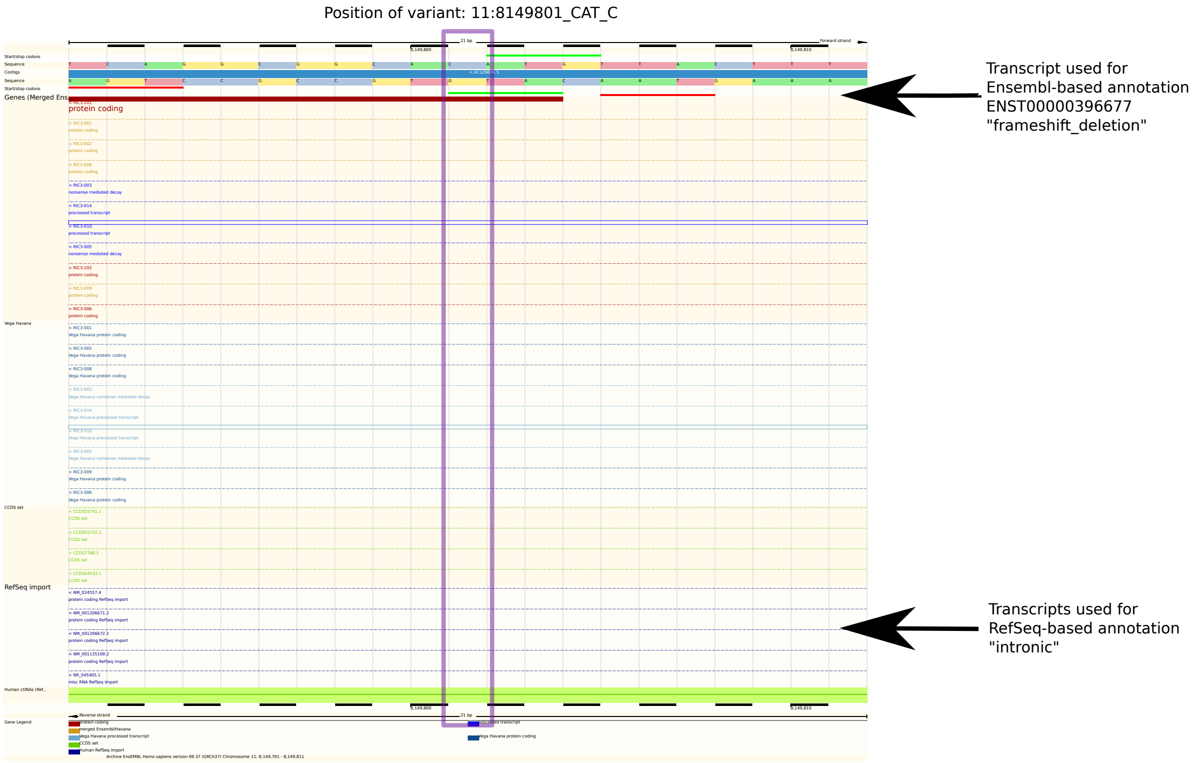

Figure 7: **Browser Image: RefSeq intronic, Ensembl frameshift deletion.** For this variant, 11:8149801_CAT_T, the different annotations from RefSeq and Ensembl are again due to the use of transcripts with different structures. At this position in the genome there are multiple RefSeq transcripts and even more Ensembl transcripts that have an intron. There is one Ensembl transcript that has coding sequence at this position, and this is the one used by Annovar for the Ensembl-based annotation. Given the transcripts used, both annotations look “correct” here. This image shows how many isoforms (i.e., transcripts) a gene can have.

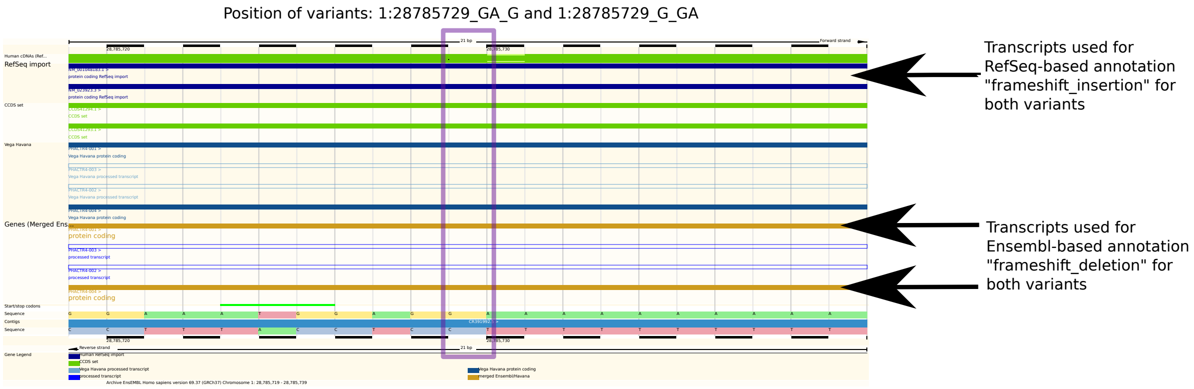

Figure 8: **Browser Image: RefSeq frameshift insertion, Ensembl frameshift deletion.** This browser image shows quite a peculiar example of annotations. At this location we observed two variants, 1:28785729_GA_G and 1:28785729_G_GA. If there is a transcript with coding sequence at this location, then we would expect the first variant to be annotated as "frameshift deletion" and the second to be annotated as "frameshift insertion". However, Annovar behaves oddly here as the two variants are *both* annotated as "frameshift insertion" when RefSeq transcripts are used and *both* annotated as "frameshift deletion" when Ensembl transcripts are used. There are 373 variants in our dataset for which this or similar behaviour occurs, but it is not at all clear why Annovar would behave in this way for these variants.

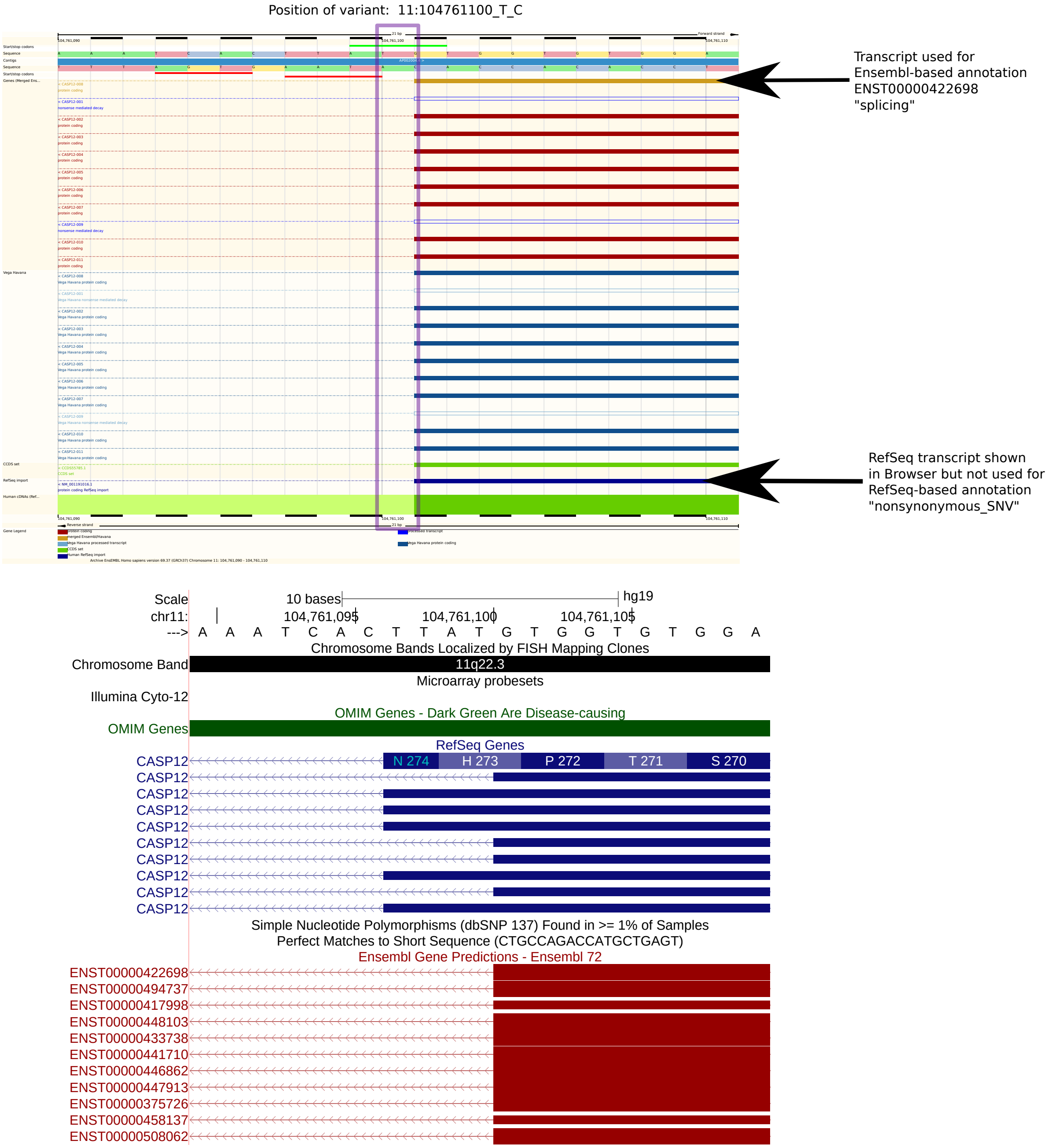

Figure 9: **Browser Image: RefSeq nonsynonymous, Ensembl splicing.** For this variant, 11:104761100_T_C, the reason for the different annotations from RefSeq and Ensembl is not apparent from looking at the Ensembl Web Browser image (top). At this position in the genome there are multiple Ensembl transcripts with the same structure and one RefSeq transcript that matches them (at least in this region shown). The "splicing" annotation given by the Ensembl transcript looks correct. Looking at the RefSeq transcript, the nonsynonymous annotation looks incorrect. However, if we look up this variant in the UCSC Web Browser (bottom), then we see that there are five RefSeq transcripts available at this position that, if used, would give an annotation of "nonsynonymous". Thus it looks like both annotations are reasonable based on the transcripts used, although this is not clear from looking only at the (archived version 69) Ensembl Web Browser.

Position of variant: 17:38064469_T_C

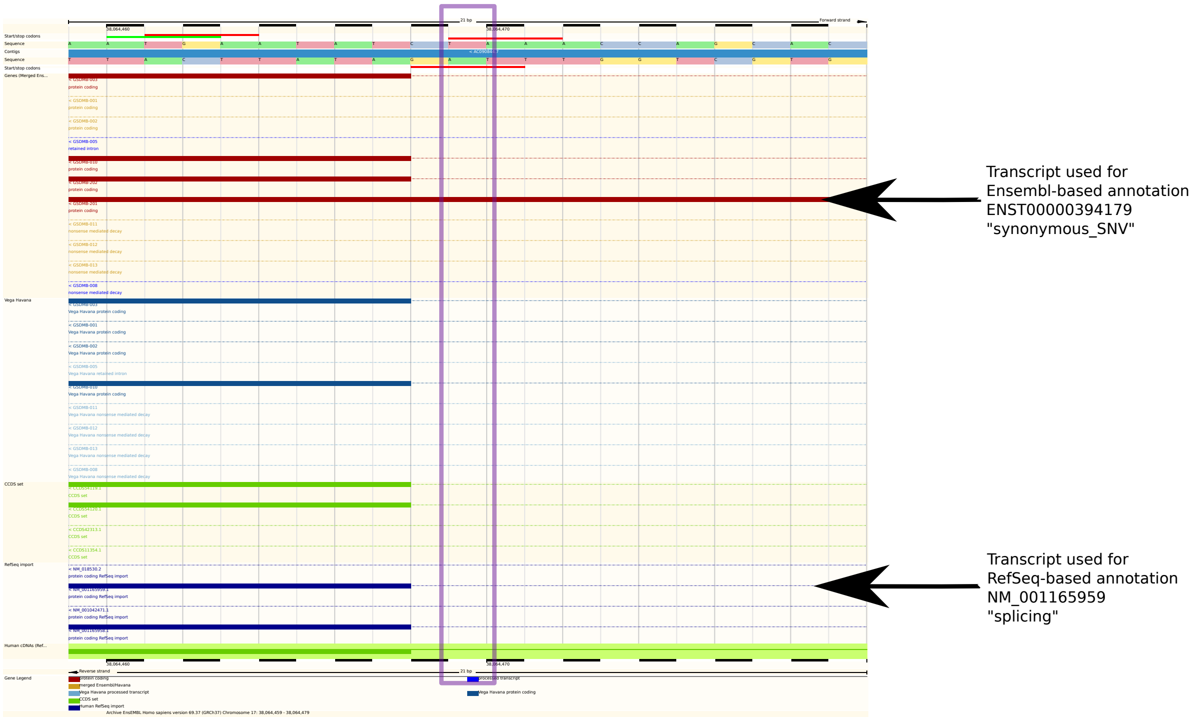

Figure 10: **Browser Image: RefSeq intronic, Ensembl frameshift deletion.** For this variant, 17:38064469_T_C, the different annotations from RefSeq and Ensembl are again due to the use of transcripts with different structures. At this position in the genome there are two RefSeq transcripts and several more Ensembl transcripts that share structure and have an intron. There is one Ensembl transcript that has coding sequence at this position, and this is the one used by Annovar for the Ensembl-based annotation. Given the transcripts used, both annotations look "correct" here.

5 Supplementary Tables

Table 1: The count for each combination of RefSeq annotation and Ensembl annotation for the 80971319 variants that are annotated by either RefSeq or Ensembl. The abbreviated column names are the same as the rownames, which indicate the different categories of annotation. The entries along the diagonal should be equal (identical categories). Ideally, all of the off-diagonal entries should be zero.

	DN	FD	FI	FS	IG	IN	nc_EX	nc_IN	nc_splicing	nc_UTR3	nc_UTR5	nc_UTR5_nc_UTR3	NFD	NFI	NFS	NS	SP	SG	SL	SY	UN	UP	UP_DN	UTR3	UTR5	UTR5_UTR3
downstream	323249	5	10	0	24905	39579	7199	36584	152	2066	15	7	1	0	2	196	49	11	5	79	28	609	9493	30145	270	25
frameshift_deletion	0	4436	0	0	7	8	84	2	1	0	0	0	2	0	0	0	0	0	0	0	3	0	0	1	3	0
frameshift_insertion	0	373	4813	7	4	14	76	3	0	0	0	0	0	0	0	0	0	0	0	0	4	1	0	0	3	0
frameshift_substitution	0	5	0	182	0	2	6	0	0	0	0	0	0	0	0	0	0	0	0	0	0	0	0	0	0	0
intergenic	481779	304	253	139	40799619	2004467	327992	5637168	7657	3544	930	9	112	94	55	8345	1371	351	37	3529	923	448820	18240	42210	9087	78
intronic	3255	866	436	220	105303	25260576	108884	1152225	2226	3178	1874	21	179	136	54	11053	29953	571	189	4209	2764	4385	192	62935	49352	828
ncRNA_exonic	4173	122	70	30	14473	10635	84152	9340	117	294	161	2	52	34	16	2161	66	120	18	862	150	2819	643	7308	2365	120
ncRNA_intronic	9639	25	10	10	329371	353656	35082	1365860	2079	1581	739	1	6	9	12	575	810	18	8	255	73	9023	1228	2083	1261	14
ncRNA_splicing	8	0	0	0	58	21	101	47	682	0	0	0	0	0	0	3	107	0	0	2	0	15	0	4	3	0
ncRNA_UTR3	195	0	0	0	324	349	189	649	13	4466	0	0	0	0	0	20	18	3	0	9	6	0	0	4465	0	6
ncRNA_UTR5	0	0	0	0	3	35	138	78	9	0	750	1	0	0	0	14	12	0	0	5	3	12	4	1	909	15
ncRNA_UTR5_ncRNA_UTR3	0	0	0	0	0	0	0	0	0	0	0	0	0	0	0	0	0	0	0	0	0	0	0	0	0	1
nonframeshift_deletion	0	46	1	67	7	3	42	0	0	0	0	0	3148	0	0	0	0	0	2	0	0	1	0	0	4	0
nonframeshift_insertion	0	72	150	35	3	12	29	6	0	0	0	0	346	2226	0	0	0	4	0	0	3	1	1	0	0	0
nonframeshift_substitution	0	9	0	49	0	0	16	0	0	0	0	0	16	0	136	0	0	0	0	0	0	0	0	0	0	0
nonsynonymous_SNV	62	495	283	532	297	308	2455	74	0	11	3	0	484	150	257	285821	17	167	83	63	95	59	15	42	122	3
splicing	1	68	21	140	35	113	217	20	110	6	35	1	17	2	83	172	12115	5	8	73	63	7	1	149	689	3
stopgain_SNV	6	79	95	203	15	17	172	8	3	0	0	0	37	11	53	158	0	13308	0	6	3	2	0	2	5	0
stoploss_SNV	1	4	0	0	0	6	1	1	19	0	0	0	0	0	3	2	1	0	463	1	0	0	0	0	1	0
synonymous_SNV	20	212	139	235	119	114	1152	30	2	7	0	1	494	131	135	2862	8	205	60	161465	44	35	6	21	64	0
unknown	2	65	91	71	46	41	2151	47	9	1	1	0	18	24	28	1777	36	72	7	920	713	0	0	67	28	0
upstream	863	27	18	3	13181	57988	11313	35689	275	41	1122	0	1	3	0	435	349	17	0	189	113	292574	7380	741	18343	63
upstream_downstream	603	0	1	0	141	1364	635	1850	7	102	68	0	0	0	0	15	11	3	0	3	1	585	9306	290	607	29
UTR3	16513	126	64	22	19497	12027	13081	4392	175	31795	23	39	13	8	4	1159	463	72	25	447	460	784	667	471894	140	365
UTR5	7	41	40	2	300	2414	2778	368	25	4	3715	19	37	16	5	823	453	32	1	343	254	2932	117	81	79397	341
UTR5_UTR3	0	0	0	0	0	0	2	0	1	37	8	5	0	0	0	1	0	1	0	3	0	0	0	2	31	201

Table 2: The count for each combination of ANNOVAR annotation and VEP annotation for the 80971319 variants that are annotated by either software tool. The rows represent VEP annotations and the columns ANNOVAR annotations. A large number of variants across many different categories show disagreement in annotations from the two annotation tools.

	.	downstream	frameshift	intergenic	intronic	ncRNA_exonic	ncRNA_intronic	ncRNA_splicing	ncRNA_UTR3	ncRNA_UTR5	nonframeshift	nonsynonymous	splicing	stopgain	stoploss	synonymous	unknown	upstream	UTR3	UTR5
3_prime_UTR_variant	133	2259	25	3325	4580	11875	101	13	46754	1	3	33	0	2	1	10	1	121	617414	9
5_prime_UTR_variant	36	21	12	704	3089	5253	108	1	0	9333	7	14	0	0	0	9	18	1124	29	160646
coding_sequence_variant	0	0	104	1	0	9	0	0	0	0	5	3	31	0	0	1	31	0	0	0
downstream_gene_variant	580	659966	0	2369212	368	187	109	0	0	0	0	0	0	0	0	0	0	4	500	0
frameshift_variant	16	2	13486	1115	94	5	1	0	0	0	421	295	68	451	25	137	518	0	18	33
incomplete_terminal_codon_variant	0	0	0	14	0	0	0	0	0	0	0	1	0	0	0	9	18	0	0	0
inframe_deletion	0	0	55	36	6	3	0	0	0	0	3262	0	1	0	0	0	31	0	0	0
inframe_insertion	22	4	229	61	6	0	0	0	0	0	2533	140	18	7	0	118	65	0	2	1
initiator_codon_variant	0	0	9	77	4	0	1	0	0	0	14	1087	0	0	0	0	5	0	0	2
intergenic_variant	5819	207	0	35209129	1963	131	1232	5	0	0	0	3	3	0	0	2	38	334	34	2
intron_variant	4487	2921	42	86568	23803629	113051	765339	59	143	3	3	124	1	1	0	38	19	3305	1265	588
mature_miRNA_variant	0	0	0	236	24	3815	0	0	0	0	0	0	0	0	0	0	0	1	0	0
missense_variant	0	24	944	19401	697	72	30	0	0	0	1891	312261	22	81	2	1073	3240	22	120	73
nc_intron_variant	2386	28375	13	809239	3906669	143084	7471911	17	110	43	0	162	2	6	3	102	4	29260	651	332
nc_splice_acceptor_variant	0	8	32	115	545	413	54	2058	3	0	12	60	641	0	0	31	4	14	56	5
nc_splice_donor_variant	0	9	23	114	639	511	84	2104	5	6	13	84	1175	0	0	31	8	31	43	56
nc_splice_region_variant	4	85	16	916	6845	7333	4555	9241	67	63	3	45	4285	1	0	758	8	174	466	435
nc_transcript_variant	0	0	0	5	0	3	0	48	0	0	0	0	0	0	0	0	0	0	0	0
non_coding_exon_variant	44	4119	28	24997	0	311319	13	7	0	0	7	294	2	10	1	134	3	4022	787	122
splice_acceptor_variant	1	0	151	11	60	41	0	0	2	2	76	127	4415	6	0	65	9	0	23	22
splice_donor_variant	1	1	236	16	101	78	4	2	1	3	36	258	7536	2	1	140	36	3	32	57
splice_region_variant	4	6	60	126	13214	395	655	5	48	95	11	16	27629	0	0	3918	80	21	829	2186
stop_gained	0	1	78	1271	31	2	0	0	0	0	63	132	3	14348	0	55	154	1	4	3
stop_lost	0	0	6	40	17	0	0	0	0	0	9	36	2	0	870	16	77	0	3	1
stop_retained_variant	0	0	1	5	2	1	0	0	0	0	0	1	0	0	2	173	17	0	0	1
synonymous_variant	0	11	270	6691	292	34	10	0	0	1	281	414	0	45	1	165643	1280	4	46	30
upstream_gene_variant	636	142357	2	2774283	874	332	234	2	0	0	0	2	5	0	0	0	39	771516	119	172

Table 3: Common-sense precedence values used to prioritise VEP annotations for comparison with Annovar annotions. Higher values indicate higher precedence for the consequence, so if a variant receives more than one annotation the consequence with the higher precedence value is reported for the comparison.

VEP Consequence	Precendence
transcript_ablation	100
splice_donor_variant	87
splice_acceptor_variant	86
stop_gained	99
frameshift_variant	85
stop_lost	95
initiator_codon_variant	75
inframe_insertion	71
inframe_deletion	70
missense_variant	65
transcript_amplification	60
splice_region_variant	63
incomplete_terminal_codon_variant	50
synonymous_variant	40
stop_retained_variant	45
coding_sequence_variant	35
mature_miRNA_variant	30
UTR5_prime_UTR_variant	26
UTR3_prime_UTR_variant	25
intron_variant	24
NMD_transcript_variant	21
non_coding_exon_variant	20
nc_transcript_variant	19
upstream_gene_variant	18
downstream_gene_variant	17
TFBS_ablation	15
TFBS_amplification	16
TF_binding_site_variant	14
regulatory_region_variant	11
regulatory_region_ablation	12
regulatory_region_amplification	13
feature_elongation	2
feature_truncation	1
intergenic_variant	0

Table 4: Annovar and VEP terms in LoF, nonsynonymous/missense and exonic categories. In some figures and tables, frameshift insertions and deletions are combined into one "frameshift" category, with similar treatment for "nonframeshift" variants.

Category	Annovar Terms	VEP Terms
Loss-of-function	"frameshift_deletion"	"frameshift_variant"
	"frameshift_insertion"	"splice_donor_variant"
	"splicing"	"splice_acceptor_variant"
	"stopgain_SNV"	"stop_gained"
	"stoploss_SNV"	"stop_lost"
		"transcript_ablation"
Missense/Nonsynonymous	"nonframeshift_deletion"	"inframe_insertion"
	"nonframeshift_insertion"	"inframe_deletion"
	"nonsynonymous_SNV"	"splice_region_variant"
		"initiator_codon_variant"
Synonymous	"synonymous_SNV"	"synonymous_variant"
		"stop_retained_variant"
Exonic	All of the above	All of the above plus
		"coding_sequence_variant"
		"incomplete_terminal_codon_variant"

Table 5: Matching rate for annotations across the range of minor allele frequencies for variants.

MAF Range	Total	Matching	Match Rate
0–1%	67313352	57701490	85.72
1–5%	5802063	4882133	84.14
5–10%	1656880	1388842	83.82
10+%	6209280	5209087	83.89

(a) Same software, different transcript set.

MAF Range	Total	Matching	Match Rate
0–1%	67327521	61819362	91.82
1–5%	5802063	5308312	91.49
5–10%	1656880	1510709	91.18
10+%	6209280	5667996	91.28

(b) Same transcript set, different software.

Table 6: Differences in annotations for variants annotated as frameshift by only one of ANNOVAR or VEP.

Consequence	Matching_Tx	Mismatching_Tx
3_prime_UTR_variant	18	7
5_prime_UTR_variant	10	2
coding_sequence_variant	102	2
inframe_deletion	37	18
inframe_insertion	217	12
initiator_codon_variant	8	1
intron_variant	9	33
missense_variant	905	39
nc_intron_variant	0	13
nc_splice_acceptor_variant	0	32
nc_splice_donor_variant	0	23
nc_splice_region_variant	5	11
non_coding_exon_variant	24	4
splice_acceptor_variant	113	38
splice_donor_variant	178	58
splice_region_variant	52	8
stop_gained	74	4
stop_lost	5	1
stop_retained_variant	1	0
synonymous_variant	257	13
upstream_gene_variant	0	2
TOTAL	2015	321

(a) Frameshift: ANNOVAR only, VEP consequences

Consequence	Matching_Tx	Mismatching_Tx
.	0	16
downstream	0	2
intergenic	0	1115
intronic	0	94
ncRNA_exonic	0	5
ncRNA_intronic	0	1
nonframeshift	394	27
nonsynonymous	283	12
splicing	28	40
stopgain	437	14
stoploss	25	0
synonymous	133	4
unknown	0	518
UTR3	0	18
UTR5	0	33
TOTAL	1300	1899

(b) Frameshift: VEP only, ANNOVAR consequences

Table 7: Differences in annotations for variants labelled as stop-gain by either ANNOVAR or VEP.

Consequence	Matching_Tx	Mismatching_Tx
3_prime_UTR_variant	0	2
frameshift_variant	437	14
inframe_insertion	7	0
intron_variant	0	1
missense_variant	77	4
nc_intron_variant	0	6
nc_splice_region_variant	1	0
non_coding_exon_variant	6	4
splice_acceptor_variant	2	4
splice_donor_variant	1	1
synonymous_variant	39	6
TOTAL	570	42

(a) Stopgain: ANNOVAR only, VEP consequences

Consequence	Matching_Tx	Mismatching_Tx
downstream	0	1
frameshift	74	4
intergenic	0	1271
intronic	0	31
ncRNA_exonic	0	2
nonframeshift	52	11
nonsynonymous	77	55
splicing	0	3
synonymous	22	33
unknown	0	154
upstream	0	1
UTR3	0	4
UTR5	0	3
TOTAL	225	1573

(b) Stopgain: VEP only, ANNOVAR consequences

Table 8: Differences in annotations for variants labelled as stop-loss by either Annovar or VEP.

Consequence	Matching_Tx	Mismatching_Tx
3_prime_UTR_variant	1	0
frameshift_variant	25	0
missense_variant	1	1
nc_intron_variant	0	3
non_coding_exon_variant	1	0
splice_donor_variant	0	1
stop_retained_variant	2	0
synonymous_variant	0	1
TOTAL	30	6

(a) Stoploss: Annovar only, VEP consequences

Consequence	Matching_Tx	Mismatching_Tx
frameshift	5	1
intergenic	0	40
intronic	0	17
nonframeshift	5	4
nonsynonymous	4	32
splicing	0	2
synonymous	2	14
unknown	0	77
UTR3	0	3
UTR5	0	1
TOTAL	16	191

(b) Stoploss: VEP only, Annovar consequences

Table 9: Differences in annotations for variants labelled as splicing by either ANNOVAR or VEP.

Consequence	Matching_Tx	Mismatching_Tx
coding_sequence_variant	28	3
frameshift_variant	28	40
inframe_deletion	0	1
inframe_insertion	9	9
intergenic_variant	0	3
intron_variant	0	1
missense_variant	1	21
nc_intron_variant	0	2
nc_splice_acceptor_variant	11	630
nc_splice_donor_variant	6	1169
nc_splice_region_variant	36	4249
non_coding_exon_variant	0	2
stop_gained	0	3
stop_lost	0	2
upstream_gene_variant	0	5
TOTAL	119	6140

(a) Splicing: ANNOVAR only, VEP consequences

Consequence	Matching_Tx	Mismatching_Tx
.	0	6
downstream	0	7
frameshift	343	104
intergenic	0	153
intronic	0	13375
ncRNA_exonic	0	514
ncRNA_intronic	0	659
ncRNA_splicing	2	5
ncRNA_UTR3	0	51
ncRNA_UTR5	0	100
nonframeshift	74	49
nonsynonymous	15	386
stopgain	3	5
stoploss	0	1
synonymous	3103	1020
unknown	0	125
upstream	0	24
UTR3	0	884
UTR5	0	2265
TOTAL	3540	19733

(b) Splicing: VEP only, ANNOVAR consequences

Table 10: Variants that get an annotation of "stop-gain" from ANNOVAR and "missense" from VEP, when the same transcripts were used for annotation. Columns give: variant ID, ANNOVAR annotation, transcript used for VEP annotation and VEP annotation, respectively.

VARID	ANNOVAR Anno	VEP Transcript	VEP Anno
1:7879404_G_C	stopgain	ENST00000377532	missense_variant
1:16258037_G_A	stopgain	ENST00000375759	missense_variant
1:17084304_C_A	stopgain	ENST00000442552	missense_variant
1:17087249_C_A	stopgain	ENST00000442552	missense_variant
1:26608873_T_A	stopgain	ENST00000374217	missense_variant
1:26608873_T_C	stopgain	ENST00000374217	missense_variant
1:26608873_T_G	stopgain	ENST00000374217	missense_variant
1:119964908_C_A	stopgain	ENST00000543831	missense_variant
1:144852405_T_A	stopgain	ENST00000369356	missense_variant
1:145323674_G_A	stopgain	ENST00000342960	missense_variant
1:150315790_A_AGTATACTAATATCTCTGC	stopgain	ENST00000324862	missense_variant
1:150315790_A_AGTATACTAATATCTCTGCCTG	stopgain	ENST00000324862	missense_variant
1:152277622_G_A	stopgain	ENST00000368799	missense_variant
1:248525486_G_A	stopgain	ENST00000366475	missense_variant
10:5202131_T_G	stopgain	ENST00000473890	missense_variant
10:33136820_A_G	stopgain	ENST00000375028	missense_variant
10:75037999_C_A	stopgain	ENST00000401621	missense_variant
11:2439425_C_A	stopgain	ENST00000533060	missense_variant
11:62600479_C_A	stopgain	ENST00000536401	missense_variant
13:25671369_G_A	stopgain	ENST00000569397	missense_variant
13:103386923_A_T	stopgain	ENST00000322527	missense_variant
13:108518712_T_C	stopgain	ENST00000375915	missense_variant
13:109550478_G_A	stopgain	ENST00000457511	missense_variant
14:22974149_T_A	stopgain	ENST00000390501	missense_variant
14:24769880_A_G	stopgain	ENST00000267425	missense_variant
14:106330067_C_G	stopgain	ENST00000488476	missense_variant
15:23406700_C_A	stopgain	ENST00000558241	missense_variant
15:62204043_G_A	stopgain	ENST00000261517	missense_variant
15:69728956_G_A	stopgain	ENST00000260363	missense_variant
16:2506986_G_A	stopgain	ENST00000397066	missense_variant
16:9017090_T_TCATTTAAAC	stopgain	ENST00000542333	missense_variant
16:89920898_C_G	stopgain	ENST00000378247	missense_variant
17:7162096_C_A	stopgain	ENST00000574993	missense_variant
17:21319873_C_A	stopgain	ENST00000331718	missense_variant
19:603749_G_A	stopgain	ENST00000251287	missense_variant
19:20973558_G_A	stopgain	ENST00000425625	missense_variant
19:22937168_C_A	stopgain	ENST00000442497	missense_variant
19:39423335_A_G	stopgain	ENST00000407800	missense_variant
2:109371388_G_C	stopgain	ENST00000283195	missense_variant
2:176965359_C_A	stopgain	ENST00000404162	missense_variant
20:25259765_G_C	stopgain	ENST00000216962	missense_variant
20:29628320_G_C	stopgain	ENST00000278882	missense_variant
20:34090263_C_A	stopgain	ENST00000342580	missense_variant
22:22516884_C_A	stopgain	ENST00000390284	missense_variant
3:38103776_G_A	stopgain	ENST00000308059	missense_variant
3:56650057_A_C	stopgain	ENST00000422222	missense_variant
3:112253057_T_C	stopgain	ENST00000402314	missense_variant
3:112648144_G_A	stopgain	ENST00000440122	missense_variant
3:155493498_G_A	stopgain	ENST00000340171	missense_variant
3:197880135_C_A	stopgain	ENST00000431569	missense_variant
5:40841573_T_C	stopgain	ENST00000381677	missense_variant
5:64868102_G_C	stopgain	ENST00000514172	missense_variant
5:96232541_G_A	stopgain	ENST00000513084	missense_variant
5:115351067_C_A	stopgain	ENST00000357872	missense_variant
5:118484997_G_C	stopgain	ENST00000539542	missense_variant
5:137217768_G_A	stopgain	ENST00000239926	missense_variant
5:176026123_A_G	stopgain	ENST00000303991	missense_variant
5:179193668_G_A	stopgain	ENST00000292599	missense_variant
6:29911227_G_C	stopgain	ENST00000396634	missense_variant
6:38800164_G_A	stopgain	ENST00000359357	missense_variant
6:99958089_A_C	stopgain	ENST00000508908	missense_variant
6:109768585_A_G	stopgain	ENST00000358807	missense_variant
6:139170553_G_A	stopgain	ENST00000367682	missense_variant
6:154567863_C_G	stopgain	ENST00000337049	missense_variant
6:158535748_A_ATATAAAATGAGATAATAC	stopgain	ENST00000435180	missense_variant
6:158535748_A_ATATAAAATGAGATAATACATT	stopgain	ENST00000435180	missense_variant
6:160113846_T_C	stopgain	ENST00000452684	missense_variant
6:161127558_G_A	stopgain	ENST00000366924	missense_variant
7:4245600_C_A	stopgain	ENST00000404826	missense_variant
7:21737781_C_G	stopgain	ENST00000328843	missense_variant
7:100385565_T_C	stopgain	ENST00000546213	missense_variant
7:100385565_T_G	stopgain	ENST00000546213	missense_variant
7:114269998_CA_CAAGA	stopgain	ENST00000452963	missense_variant
7:142569476_C_A	stopgain	ENST00000359396	missense_variant
8:25341499_G_A	stopgain	ENST00000380665	missense_variant
8:27622762_G_A	stopgain	ENST00000524084	missense_variant
9:74489737_G_A	stopgain	ENST00000377041	missense_variant

References

1. Pruitt K, Brown G, Tatusova T, Maglott D: **The Reference Sequence (RefSeq) Database**. In *The NCBI Handbook [Internet]*. Edited by McEntyre J, Ostell J, Bethesda (MD): National Center for Biotechnology Information (US) 2002 [Updated 2012 Apr 6]:Chapter 18. [Available from: http://www.ncbi.nlm.nih.gov/books/NBK21091/].
2. Ensembl: **Ensembl Gene Set** 2013, [http://www.ensembl.org/info/genome/genebuild/genome_annotation.html].
3. Pruitt K, Brown G: **RefSeq Frequently Asked Questions (FAQ)** 2013, [http://www.ncbi.nlm.nih.gov/books/NBK50679/].
4. National Center for Biotechnology Information: **RefSeq: NCBI Reference Sequence Database** 2013, [http://www.ncbi.nlm.nih.gov/refseq/].
5. Ensembl: **Ensembl** 2013, [http://www.ensembl.org/index.html].
